# Exosomes as nanocarriers for brain-targeted delivery of therapeutic nucleic acids: advances and challenges

**DOI:** 10.1186/s12951-025-03528-2

**Published:** 2025-06-18

**Authors:** Nima Sanadgol, Mohsen Abedi, Masoud Hashemzaei, Zahra Kamran, Roghayeh Khalseh, Cordian Beyer, Clara Voelz

**Affiliations:** 1 Institute of Neuroanatomy, RWTH University Hospital Aachen, Aachen, Germany; 2 Department of Advanced Technologies, North Khorasan University of Medical Sciences, Bojnurd, Iran; 3 Pharmaceutical Sciences Research Center, Shiraz University of Medical Sciences, Shiraz, Iran; 4 Department of Medicine, Isfahan University of Medical Sciences, Esfahān, Iran; 5 Hannover Medical School, Institute of Functional and Applied Anatomy, Hannover, Germany

**Keywords:** BBB, Exosomes, Neurodegeneration, Neuroinflammation, Neuropharmacology

## Abstract

Recent advancements in gene expression modulation and RNA delivery systems have underscored the immense potential of nucleic acid-based therapies (NA-BTs) in biological research. However, the blood–brain barrier (BBB), a crucial regulatory structure that safeguards brain function, presents a significant obstacle to the delivery of drugs to glial cells and neurons. The BBB tightly regulates the movement of substances from the bloodstream into the brain, permitting only small molecules to pass through. This selective permeability poses a significant challenge for effective therapeutic delivery, especially in the case of NA-BTs. Extracellular vesicles, particularly exosomes, are recognized as valuable reservoirs of potential biomarkers and therapeutic targets. They are also gaining significant attention as innovative drug and nucleic acid delivery (NAD) carriers. Their unique ability to safeguard and transport genetic material, inherent biocompatibility, and capacity to traverse physiological barriers highlight their potential as drug carriers. This review provides a comprehensive overview of current strategies to enhance NAD to the brain, focusing on the emerging potential of exosomes as biocompatible and efficient nanocarriers. It synthesizes recent advances in the use of exosomes for NA-BTs in neurological disorders, comparing their advantages with those of conventional nanodelivery systems and cell-based therapies. Additionally, the review highlights innovative exosome engineering approaches to improve brain-targeted delivery, addresses key methodological limitations such as variability in cargo content, and proposes solutions to enhance standardization and safety. Collectively, these insights highlight the translational potential of exosomes and offer a novel perspective on bridging the gap between fundamental research and clinical application.

**Graphical Abstract:**

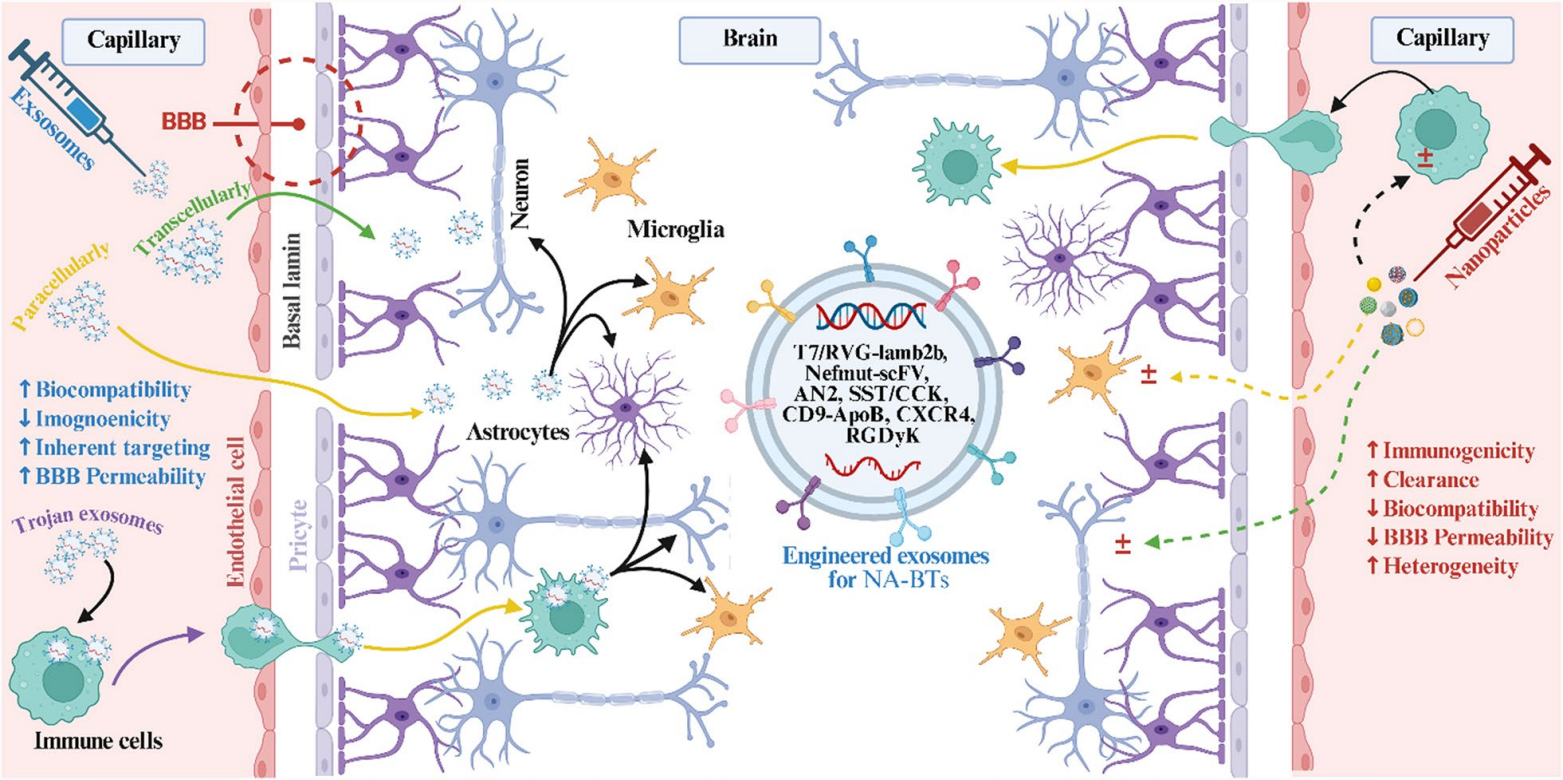

## Introduction

Central nervous system (CNS) diseases are highly significant due to their impact on one of the most crucial systems in the human body. The CNS, encompassing the brain and spinal cord, is essential for controlling and coordinating various bodily functions. Unfortunately, effective treatments for these conditions remain challenging to develop. A major obstacle is the difficulty in delivering drugs to their intended targets within the brain. For a drug to be effective, it must cross the BBB at a concentration sufficient to interact with its target [1]. The BBB is a complex physiological barrier consisting of brain capillary endothelial cells with tight junctions, receptors, enzymes, and transporters. The BBB tightly regulates the entry of molecules into the brain, safeguarding it from harmful substances while permitting the controlled transport of essential nutrients across endothelial cell membranes. However, this critical function can be compromised under certain pathological conditions. For example, in multiple sclerosis (MS), the increased permeability of the BBB enables immune cells to infiltrate the brain, thereby promoting the development of neuroinflammation [136]. Although this protective function is vital, it presents significant challenges for effective drug delivery to the brain. The endothelial cells forming the BBB have luminal and abluminal membranes facing the bloodstream and the brain, respectively. The selective permeability of the BBB is attributed to specific structural components, such as adhesion molecules and tight junctions between endothelial cells [173]. Despite advances in understanding the CNS and its disorders, treatment challenges persist, leaving a growing burden of disease. This review addresses a critical gap in the current literature by providing a focused and integrative analysis of exosomes as delivery vehicles for nucleic acid-based therapies (NA-BTs) to the brain, an area often fragmented across studies with varying methodologies and clinical relevance. Rather than reiterating exosomes as generic carriers, we offer a comparative perspective on their advantages over traditional nanocarriers and cell-based therapies, highlight innovative engineering strategies for enhancing brain-specific delivery, and critically assess methodological limitations such as variability in endogenous cargo and isolation techniques. To strengthen standardization and safety, we propose solutions including high-throughput omics approaches and the use of “empty” exosomes for precise cargo loading. By combining critical evaluation with forward-looking insights, this review aims to bridge the gap between basic exosome research and its clinical translation, positioning exosomes as a promising and distinct platform for future neurotherapeutic development.

## Nucleic acid-based therapies (NA-BTs) for neurologic disorders

Nucleic acid medications offer advantages over traditional drugs by targeting genes and utilizing Watson–Crick base pairing for precise binding. These drugs can reach therapeutic sites inaccessible to conventional drugs, targeting a broader range of targets beyond cell surface receptors. Nucleic acid drugs (NA-BTs) benefit from a faster development timeline due to easy access to target gene sequences and provide sustained therapeutic effects with their extended half-life. Major types include small interfering RNAs (siRNAs), microRNAs (miRNAs), and ASOs that inhibit RNA, while plasmid DNAs (pDNAs), peptide nucleic acids (PNAs), and messenger RNAs (mRNAs) enhance gene expression [106]. The complexity of brain tumor NA-BTs arises from various factors, particularly the genetic heterogeneity and diverse mutation profiles of tumors, which hinder the identification of consistent, broadly applicable therapeutic targets. Additionally, unmodified nucleic acids face significant hurdles such as high immunogenicity, poor stability, rapid degradation by nucleases, and low accumulation at tumor sites due to biological barriers like the blood–brain and blood-tumor barriers [45]. To overcome these obstacles, effective delivery systems are essential for enhancing nucleic acid transport and stability. The CRISPR/Cas system further allows for the precise enhancement, suppression, and rectification of target gene expression [106]. In contrast, pDNAs and mRNAs are used to increase the expression of target genes. This section focuses on the mechanisms of therapy and design of NA-BTs for neuroprotection.

### Therapeutic siRNAs and miRNAs

RNA interference (RNAi) is a mechanism that uses small RNA molecules, including siRNAs and miRNAs, to suppress gene expression by degrading or inhibiting mRNA in the cytoplasm of target cells [113] (Table 1). The enzyme Dicer, a specialized RNase III-like enzyme, processes double-stranded RNA (dsRNA) into siRNAs, which then integrate into the RNA-induced silencing complex (RISC). Within RISC, Argonaute 2 (AGO2) cleaves the sense strand of the siRNA, enabling the antisense strand to direct RISC to the corresponding mRNA, thereby inducing gene silencing [193]. Short hairpin RNAs (shRNAs) function in a similar way to synthetic siRNAs [51]. Endogenously produced miRNAs undergo transcription into primary miRNA, cleavage into precursor miRNA by Drosha, and final processing into mature miRNAs by Dicer [47]. Synthetic siRNA drugs are engineered to target specific genes and bind directly to RISC, leveraging the endogenous RNAi pathway to silence genes. Optimizing the sequence of siRNAs is essential to enhance gene silencing efficacy while minimizing off-target effects. These sequences typically range from 21 to 23 nucleotides in length, and although longer sequences can improve silencing, they may also activate the interferon pathway, leading to off-target mRNA degradation and apoptosis [108]. Synthetic miRNAs are also designed to mimic endogenous miRNAs, with double-stranded versions proving more effective. Unlike siRNAs, which rely on perfect base pairing of the antisense strand to cleave target mRNAs, miRNAs usually operate through a different mechanism: after the sense strand is removed, the antisense strand binds to target mRNAs with partial or imperfect complementarity, leading to translational repression or mRNA destabilization rather than direct cleavage. This allows miRNAs to regulate gene expression more broadly, often by suppressing translation rather than cutting the mRNA directly [140]. However, delivering miRNAs in vivo poses challenges, including nuclease degradation and immune activation. To enhance the therapeutic efficacy and stability of siRNAs and miRNAs, range of genetic (or chemical) modifications are commonly employed. Among the most widely used are 2′-O-methyl (2′-OMe) and 2′-fluoro (2′-F) modifications, which substitute the 2′ hydroxyl group on the ribose sugar to improve nuclease resistance, reduce immunogenicity, and increase binding affinity [142]. Phosphorothioate (PST) linkages, in which a sulfur atom replaces a non-bridging oxygen in the phosphate backbone, further enhance resistance to exonucleases and improve pharmacokinetic profiles. Locked nucleic acids (LNAs), characterized by a methylene bridge that locks the ribose conformation, offer increased thermal stability and specificity for the target RNA. Additionally, 5′-end modifications on the guide strand help prevent degradation and support efficient loading into the RISC, which is essential for gene silencing [33, 82]. To improve tissue targeting and cellular uptake, siRNAs and miRNAs are also frequently conjugated to molecules like cholesterol or N-acetylgalactosamine, the latter being especially effective for liver-targeted delivery(TD) [119]. These chemical strategies are often used in combination to optimize siRNA-based therapeutics for clinical application [4].

**Table 1:** Overview of key nucleic acid-based therapies

Type	Definition	Delivery system	Mechanism of action	Refs
siRNAs	~20–25 bp of double-stranded (ds) nucleotides	LNPs, polymeric carriers, viral vectors, CPPs, GalNAc conjugated	↑mRNA degradation and gene silencing	Zou et al. [193]
ASOs	~5–40 bp of single-stranded (ss) (DNA/RNA nucleotide)	Self-delivery, LNPs, CPPs, polymeric carriers	Degradation of target RNA or block RNA processing	Dhuri et al. [41]
miRNAs	Mimic: ~21–25 bp of dsRNA nucleotides	LNPs, polymeric carriers, viral vectors, aptamer-conjugated	↑Function of endogenous miRNAs	Shang et al. [140]
	AntimiRs: ~21–25 bp of ssRNA		↓Function of endogenous miRNAs	
mRNAs	Small or large ssRNA with mRNA structure	LNPs, polymeric carriers, CPPs, hydrogels	↑Exogenous or endogenous proteins	Yang et al. [180]
Aptamers	~20–80 bp of ss nucleic acids (DNA/RNA) or small peptides	Self-delivery, LNPs, polymeric carriers, siRNA chimeras	Blocking or modulating molecules’ biological activity	Doherty et al. [39]
PNAs	~10–25 bp synthetic DNA/RNA (sugar-phosphate is replaced by a backbone made of AEG)	CPPs, endocytosis, LNPs, polymeric carriers	↓Gene expression via preventing transcription or translation	Montazersaheb et al. [116]
DNA	~15–30 bp short DNA constructs	Viral vectors, electroporation, microinjection, LNPs, polymeric carriers	Produce shRNAs that degrade mRNAs or employ DNA templates for systems like TALENs	Li et al. [88] and Sussman et al. [152]
	>1 kb of circular DNA vectors (plasmids) <5 kb of ssDNA vectors (AAV)		Adding functional copies of defective genes and encoding proteins in target cells	
	~2–4 kb compact DNA constructs (Mini-circle DNA)		↑Transfection efficiency and lower immunogenicity compared to plasmids	
DNAzymes	25–40 bp ssDNA synthesized using the SELEX technique	LNPs, polymeric carriers, electroporation, CPPs	Catalytic cleaving of specific RNA targets in the presence of Mg^2^⁺ or Pb^2^⁺ as cofactors	Larcher et al. [84]
CRISPR/Cas systems	DNA targeting (~20–25 bp gRNA, ~80 tracrRNA, ~100 sgRNA), RNA targeting (~25–30 bp, crRNA)	Viral vectors, electroporation, microinjection, LNPs	Introducing precise double-stranded breaks at targeted DNA sequences or degrading RNA molecules	Alaa et al. [6]

*PNAs* peptide nucleic acids, *CRISPR* clustered regularly interspaced short palindromic repeats, *TALENs* transcription activator-like effector nucleases, *shRNAs* short hairpin RNAs, *SELEX* systematic evolution of ligands by exponential enrichment, *TALENs* transcription activator-like effector nucleases, *AEG N*-(2-aminoethyl) glycine units, *CPPS* cell-penetrating peptides, *LNPs* lipid nanoparticles, *PEI* polyethyleneimine, *GalNAc N*-acetylgalactosamine

### Therapeutic ASOs

Antisense oligonucleotides (ASOs) are single-stranded DNA-like oligonucleotide drugs typically about 20 nucleotides long (Table 1). These therapeutics target receptor RNAs, including mRNAs, ribosomal RNAs (rRNAs), and non-coding RNAs (ncRNAs), reflecting advances in RNA biology [41]. ASOs operate through two main mechanisms: occupancy-mediated degradation (RNase H-competent) and occupancy-only mechanisms (steric block). Steric block ASOs manipulate gene expression by blocking translation, disrupting upstream open reading frames (uORFs), preventing nonsense-mediated decay (NMD), and modulating polyadenylation signals [37]. ASOs are chemically modified to enhance their stability, binding affinity, and therapeutic performance. One of the most common modifications is the incorporation of PS linkages, improving resistance to nuclease degradation and enhancing cellular uptake [111]. Sugar modifications such as 2′-OMe, 2′-F, and 2′-O-methoxyethyl (2′-MOE) substitutions increase target RNA affinity and Further protect ASOs from enzymatic breakdown. LNAs are also commonly used to enhance thermal stability and binding strength. Additionally, end-capping at the 3′ or 5′ termini can provide extra protection against exonucleases [157]. Moreover, ligand conjugation, such as N-acetylgalactosamine (GalNAc), enhances ASO uptake into diseased cells via specific receptor interactions, like the asialoglycoprotein receptor [118]. These modifications are often strategically combined in ASO designs, which consist of a central DNA core flanked by modified nucleotides, enabling RNase H-mediated cleavage of the target RNA while ensuring overall structural stability and reduced immunogenicity, making ASOs a robust platform for therapeutic gene silencing. Nusinersen (Spinraza), a Food and Drug Administration (FDA)-approved treatment for spinal muscular atrophy, exemplifies the occupancy-only mechanism [38]. Combining ASOs targeting the 5′ UTR with splice-switching oligonucleotides (SSOs) can enhance survival motor neuron protein levels, offering greater efficacy than SSOs alone. ASOs can be contrasted with the rigid, double-stranded siRNAs, which use distinct cleavage mechanisms for target RNA [32]. Moreover, steric block oligonucleotides, a specialized class of ASOs, exert their effects by binding to specific regions of target RNA transcripts without inducing their degradation. Instead of activating RNase H or other cleavage pathways, these oligonucleotides physically obstruct access of the splicing machinery or ribosomes, thereby modulating RNA processing or translation. This mechanism allows for precise regulation of gene expression, such as altering splicing patterns or preventing translation, without altering RNA stability [145]. To date, three splice-switching ASOs, including eteplirsen, golodirsen, and nusinersen, have received FDA approval [137]. Advanced oligonucleotide therapies include adenosine deaminase acting on RNA (ADAR)-oligonucleotides, designed to bind specific brain mRNAs and recruit the enzyme, which catalyzes adenosine-to-inosine RNA editing. Single-stranded RNA-binding molecules (10–35 kDa) use molecular pairing to recruit ADAR, expanding therapeutic potential in RNA editing for CNS conditions. This editing is crucial for brain development and function and is implicated in neurological and psychiatric disorders [124, 181]. While ASOs have shown considerable success in treating viral infections and metabolic disorders, their application in neurological disorders remains limited and challenging.

### Therapeutic mRNAs

As research progresses, mRNA-based therapeutics emerge as a transformative approach for preventing and treating neurodegenerative diseases (NDDs) and brain tumors (Table 1). Their ability to bypass limitations associated with other therapies while offering enhanced precision and control positions mRNA therapy as a promising strategy for addressing challenging brain disorders [180]. To enhance the stability, translational efficiency, and therapeutic potential of mRNAs, several chemical modifications are commonly employed. One key strategy involves modifying the 5′ cap structure using anti-reverse cap analogs (ARCAs), which improve mRNA translation and protect against exonuclease degradation. Additionally, nucleotide modifications such as pseudouridine (Ψ), N1-methylpseudouridine (m1Ψ), 5-methylcytidine (m5C), and 2-thiouridine (s2U) are widely used to reduce innate immune responses, increase mRNA stability, and boost protein expression by evading recognition by toll-like receptors (e.g., TLR7/8). Codon optimization, which replaces codons with synonymous ones matching the host’s abundant tRNAs, further enhances translational efficiency. Engineering the untranslated regions (UTRs), particularly incorporating stabilizing sequences in the 5′ and 3′ UTRs, also contributes to improved mRNA stability and longevity in cells. Lastly, optimizing the poly(A) tail length enhances protection against degradation and promotes efficient translation [103]. Together, these modifications are critical in the design of therapeutic mRNAs, including those used in vaccines and gene therapies, ensuring they are stable, non-immunogenic, and highly functional. Unlike pDNA, mRNA does not integrate into the genome, mitigating the risk of oncogenic mutations. However, mRNA delivery faces challenges due to its anionic nature and susceptibility to degradation. Delivery systems such as lipids and polymers are employed to overcome these barriers. Lipid nanoparticles (LNPs), which demonstrated success in COVID-19 vaccines like mRNA-1273 and BNT162b, effectively encapsulate mRNA, protect it from degradation, and facilitate its cellular delivery for translation [171]. Synthetic LNPs typically consist of four components: ionizable lipids, helper lipids, cholesterol, and PEG-lipids. Ionizable lipids enable mRNA encapsulation and Endosomal Escape via pKa tuning. Helper lipids like distearoylphosphatidylcholine or dioleoylphosphatidylethanolamine support structural stability, while Cholesterol enhances membrane fusion, particle rigidity, and delivery efficiency. PEG-lipids improve circulation time and prevent aggregation [80]. One study presents a novel system combining two key components: poly (β amino esters) polymers (PBAEPs) and AI-optimized mRNA [87]. PBAEPs enable efficient delivery of brain-derived neurotrophic factor (BDNF) mRNA to the brain and spinal cord through catheter-based ventricle pumping. Additionally, the 3′ untranslated region (3′UTR) of the mRNA was modified with a neuron-specific microRNA (miRNA) targeting sequence to limit BDNF protein expression in neurons, mitigating risks like overexcitation and seizures. mRNA-based therapeutics also enable the transient expression of nucleases, including CRISPR-Cas systems, allowing short-term and precise gene editing. This approach avoids the longer-term risks associated with DNA-based therapies, such as persistent nuclease activity and increased off-target effects [79]. Delivery challenges are being addressed through innovative methods such as co-delivering mRNA and single-guide RNA (sgRNA) in nanoparticles, using adeno-associated viruses (AAVs) for sgRNA, or leveraging compact nucleases like Cas12j to package both Cas enzymes and sgRNA into one system [121].

### Therapeutic aptamers

Therapeutic aptamers are single-stranded oligonucleotides that adopt unique, sequence-specific three-dimensional structures, allowing them to bind their molecular targets with high specificity and affinity (Table 1). These aptamers function as “chemical antibodies,” modulating the activity of proteins, peptides, or small molecules, making them promising candidates for treating a variety of disorders [2]. In the field of neurotherapeutics, aptamers offer several advantages, including low immunogenicity, ease of chemical synthesis, and the potential to cross the BBB when appropriately engineered [39]. Researchers have investigated their use in reducing neuroinflammation by targeting pro-inflammatory cytokines, as well as their potential as therapeutic delivery vehicles for drugs or imaging agents to the CNS [81]. Chemical modifications are essential for enhancing the therapeutic potential of aptamers by improving their stability, bioavailability, and target specificity. Unmodified aptamers are prone to rapid degradation by nucleases in biological environments, Limiting their clinical utility. To address this, several chemical strategies have been developed. Modifications to the sugar backbone, such as 2′-fluoro (2′-*F*), 2′-*O*-methyl (2′-OMe), and 2′-amino (2′-NH_2_), are commonly used to protect RNA aptamers from enzymatic degradation. The incorporation of PST linkages, where a non-bridging oxygen in the phosphate backbone is replaced by sulfur, further enhances resistance to exonucleases. Aptamers composed of L-ribose-based nucleotides, rather than the naturally occurring D-ribose configuration, are known as Spiegelmers. These mirror-image oligonucleotides exhibit high resistance to nuclease degradation due to their unnatural chirality, which prevents recognition and cleavage by cellular enzymes. As a result, Spiegelmers offer enhanced stability in biological fluids, making them promising candidates for therapeutic applications, particularly in environments where conventional aptamers would be rapidly degraded. Additionally, they retain high target specificity and binding affinity, often comparable to or exceeding that of their D-form counterparts, while also demonstrating reduced immunogenicity, further supporting their potential in clinical use [166]. Other approaches include PEGylation, which prolongs circulation time by increasing molecular weight and reducing Renal clearance, and conjugation with Lipids or other molecules to enhance cellular uptake. End-capping at the 3′ and 5′ ends and incorporation of modified bases also improve nuclease resistance and binding affinity [2]. Collectively, these chemical modifications transform aptamers into robust and versatile tools for therapeutic applications, particularly in targeted drug delivery and molecular imaging. With their versatile properties and the development of bioconjugation strategies, aptamers show great promise in overcoming challenges related to precision targeting and developing effective therapies for complex neurological conditions.

### Therapeutic PNAs

The PNAs are synthetic analogs of DNA in which the natural sugar-phosphate backbone is replaced by a peptide-like structure, N-(2-aminoethyl) glycine, which enhances their biostability and affinity for complementary nucleic acids [116] (Table 1). These unique properties, such as high specificity, low immunogenicity, and the ability to bind tightly to target DNA or RNA sequences, make PNAs powerful tools in therapeutic applications. PNAs are inherently stable due to their peptide-like backbone, but several chemical modifications are commonly applied to enhance their functionality for therapeutic use. One of the most significant modifications is γ-modification, which involves adding side chains like miniPEG at the gamma position to enhance both water solubility and binding affinity. Conjugation with cell-penetrating peptides (CPPs) like TAT or penetratin enhances cellular uptake, a major limitation of unmodified PNAs. Additionally, lipid or PEG conjugation is used to improve membrane permeability and pharmacokinetics. Base modifications, including methylated or locked bases, increase duplex stability and target specificity, while terminal modifications such as biotin or fluorophores allow for detection or TD [146]. These combined strategies significantly improve the therapeutic potential of PNAs by boosting their stability, bioavailability, and sequence-specific interaction with nucleic acids. In neuroscience, PNAs hold considerable promise for addressing genetic disorders of the nervous system, including conditions like Huntington’s disease and Duchenne muscular dystrophy, by correcting point mutations at the genetic level [125]. A significant advantage of PNAs is their ability to cross cellular membranes and specifically target DNA or RNA sequences within cells, including those within the CNS, making them a viable approach for treating neurodegenerative diseases. Furthermore, PNA-based strategies are being explored to modulate gene expression or silence disease-causing genes, providing hope for future therapeutic interventions in a wide range of human disorders [54].

### Therapeutic DNAs

Therapeutic DNA molecules, whether synthetic or naturally occurring, are designed for introduction into the body to treat a range of conditions, including genetic disorders, altered gene expression, and immune modulation (Table 1). These DNA-based therapies encompass gene therapy, DNA vaccines, and the use of DNA as a template to produce therapeutic proteins. Gene therapy approaches utilizing AAV or pDNA have been shown to function by replacing defective genes, restoring absent ones, or silencing the expression of harmful genes in individuals with chronic diseases [152].

The pDNA, a circular DNA molecule ranging from 2000 to 20,000 base pairs, is commonly used as a gene vector in DNA vaccines and gene therapies. It delivers genes encoding specific proteins to induce immune responses or treat genetic disorders. Compared to mRNA, pDNA offers advantages in terms of stability, cost-effectiveness, and transportability. However, its larger size can reduce delivery efficiency and restrict its use in some cases [105]. A significant development in therapeutic DNA has been the creation of DNA vaccines, which use genetic material to stimulate immune responses against specific pathogens, as seen in the COVID-19 vaccine efforts [143]. Furthermore, DNA-based therapies have the potential to deliver gene-editing tools like CRISPR/Cas9 directly into patient cells to correct genetic mutations [88]. Therapeutic DNA molecules are often chemically modified to enhance their stability, bioavailability, and therapeutic function, especially to withstand enzymatic degradation and improve delivery efficiency. A widely used modification involves incorporating a PST backbone, which greatly enhances resistance to nuclease degradation and extends the molecule’s circulation time in the body. 2′-O-M and 2′-F sugar modifications are also frequently used to stabilize the DNA structure and reduce immune recognition. For improved cellular uptake, DNA can be conjugated with lipophilic groups such as cholesterol or PEGylated with polyethylene glycol to increase solubility, reduce renal clearance, and minimize immunogenicity. Incorporation of LNAs also enhances hybridization affinity and thermal stability of the DNA strands. Additionally, strategies to methylate or suppress immunostimulatory CpG motifs help reduce unintended activation of the innate immune system [167]. These chemical modifications, often used in combination, are essential for transforming native DNA sequences into robust, clinically viable therapeutic agents with enhanced functionality and safety profiles. Despite the considerable promise of therapeutic DNA, challenges such as delivery efficiency, immune responses, and maintaining long-term expression continue to hinder progress. Nevertheless, advances in gene delivery vectors-such as LNPs and viral vectors-are improving the safety and effectiveness of DNA-based therapies, with increasing success in clinical trials.

### DNAzymes

DNAzymes (or deoxyribozymes) are synthetic single-stranded DNA molecules that possess catalytic activity, capable of cleaving specific RNA targets. As NA-BTs, they offer a highly selective means of gene silencing by binding to complementary RNA sequences and cleaving them at defined sites, thereby inhibiting gene expression (Table 1). Unlike siRNA or ASOs, DNAzymes Function through a catalytic mechanism without relying on endogenous cellular machinery, making them attractive for targeting mRNAs involved in disease processes, especially in cancers, viral infections, and neurological disorders. To improve their therapeutic potential, DNAzymes are commonly modified to enhance stability and cellular uptake. The most widely used chemical modifications include PS backbone modifications, 3′-end capping, LNAs, and 2′-O-M modifications [84]. DNAzymes designed to target and cleave repeated cytosine–adenine–guanine (CAG) sequences present in polyglutamine (polyQ) neurodegenerative diseases have shown potential to enhance cell viability without disrupting mitochondrial Function. It has been demonstrated that DNAzymes retain their catalytic activity in the mouse brain for at least 1 month post-delivery. In a Spinocerebellar Ataxia Type 3 (SCA3) mouse model, these DNAzymes markedly reduced levels of the pathogenic high-molecular-weight ATXN3 protein [191]. These findings suggest that DNAzymes represent a promising RNA-silencing strategy for the treatment of multiple polyQ-related disorders. For cellular delivery, DNAzymes are typically incorporated into nanocarrier systems after conjugation with cholesterol or PEG to improve serum stability and pharmacokinetics, which protects them from degradation and enhances target specificity [175]. These combined strategies (chemical modification and advanced delivery systems) enable DNAzymes to act as stable, potent, and specific gene-silencing tools for therapeutic applications.

### CRISPR/Cas systems

For therapeutic applications, the CRISPR/Cas9 system (originally derived from *Streptococcus pyogenes*) is widely utilized for precise genome editing. CRISPR/Cas9 functions by using a single guide RNA (sgRNA) to direct the Cas9 endonuclease to a specific DNA sequence, where it introduces a double-strand break (DSB) (Table 1). This break is then repaired by cellular DNA repair mechanisms (non-homologous end joining, or homology-directed repair) [117]. For therapeutic purposes, CRISPR/Cas9 has been employed to correct disease-causing mutations in monogenic disorders, disrupt pathogenic genes, or insert therapeutic sequences in a site-specific manner [106]. Advances in Cas9 engineering have led to the development of high-fidelity variants (e.g., eSpCas9, SpCas9-HF1) that minimize off-target effects. Moreover, catalytically inactive forms of Cas9 (dCas9) have been fused to transcriptional regulators, epigenetic modifiers, or base editors to modulate gene expression, DNA methylation, or perform single-base editing without creating DSBs [6].

For RNA-based therapeutics, specific CRISPR/Cas systems-particularly the type VI Cas13 family (Cas13a, Cas13b, Cas13d, and Cas13X/Y)-have been adapted to target single-stranded RNA rather than DNA, enabling gene regulation without introducing permanent genomic changes (Table 1). Among these, Cas13d (RfxCas13d) is especially promising due to its compact size, high specificity, and efficiency in vivo, making it suitable for therapeutic RNA knockdown and viral RNA degradation [183]. Catalytically inactivated versions (dCas13) have been repurposed as programmable RNA-binding platforms and used for RNA imaging, splicing modulation, and epitranscriptomic editing-often by Fusing with enzymes such as Adenosine Deaminase Acting on RNA 2 to mediate adenosine-to-inosine (A-to-I) RNA editing [11]. More recently, Cas7–11, part of the type III-E CRISPR system, has emerged as a highly specific RNA-targeting nuclease with reduced collateral activity compared to Cas13, offering an alternative for precise RNA modulation [186]. Delivery of these CRISPR components into target cells is achieved through viral vectors (AAV, lentivirus), LNPs, electroporation, or exosome-based systems. Collectively, these RNA-targeting CRISPR systems offer versatile and precise platforms for therapeutic intervention in diseases involving aberrant RNA expression, particularly in the CNS.

## Blood–brain barrier structure and function

**Fig. 1:**
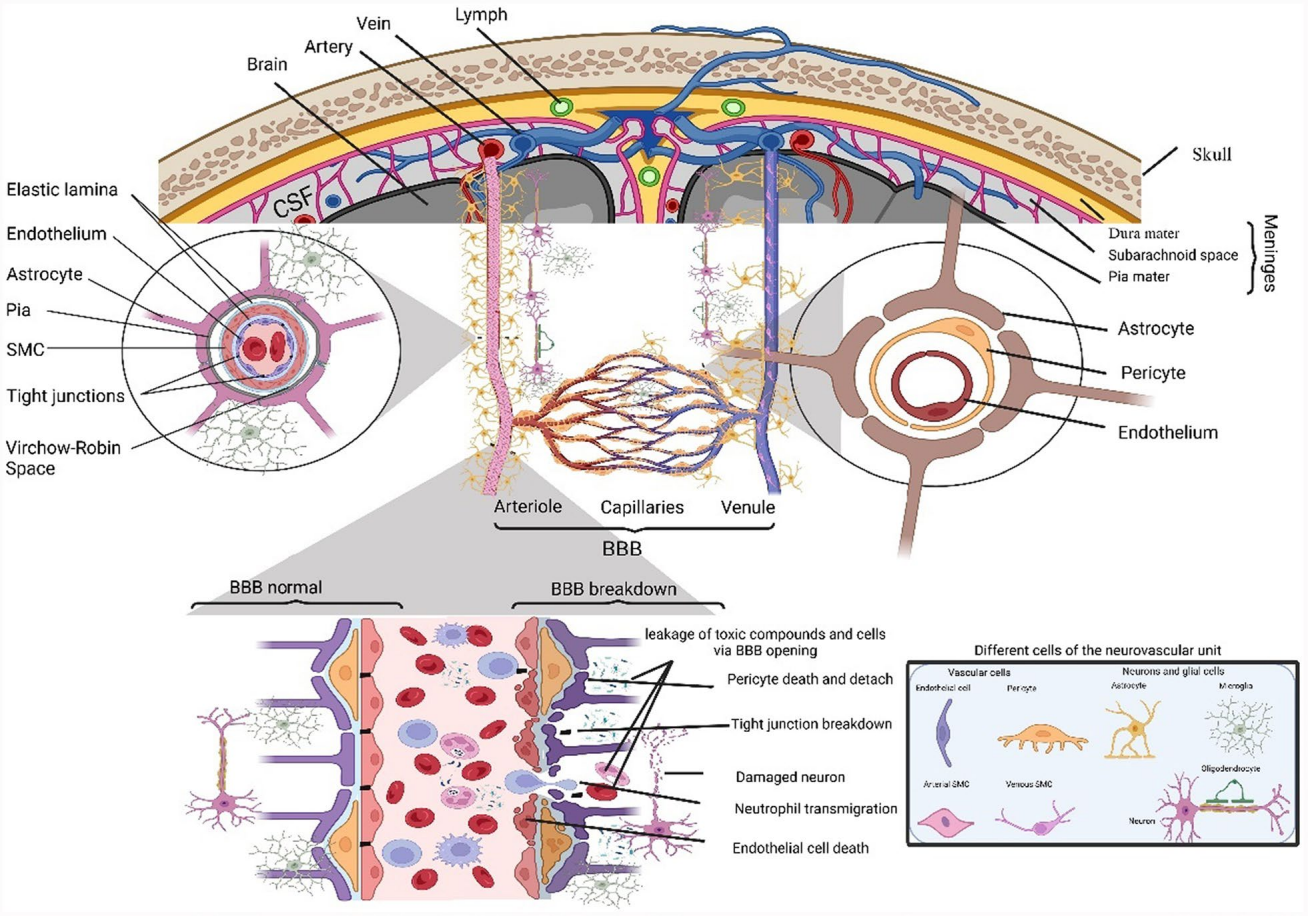
The structure of the neurovascular section. The neurovascular unit (NVU) comprises neurons, glial cells (astrocytes, microglia, oligodendrocytes), and vascular cells (endothelial cells, pericytes, and smooth muscle cells (SMCs)). The NVU’s structure varies along the vascular tree due to differences in the molecular expression of endothelial and mural cells. Endothelial cells form the inner vascular wall at the penetrating arteries, separated from SMCs by the basement membrane. The Virchow–Robin space lies between the pia and the glia limitans. At the arteriolar level, SMCs are organized in a single layer, whereas at the capillary level, pericytes and endothelial cells share a common basement membrane, which is further enveloped by the endfeet of astrocytes. Neurons innervate astrocytes, pericytes, and SMCs. The blood–brain barrier (BBB) is a monolayer of tightly sealed endothelial cells with low permeability, centrally located within the NVU. Cells within the NVU are crucial for angiogenesis, neurogenesis, BBB integrity, cerebral blood flow regulation, extracellular matrix interactions, and neurotransmitter clearance. *Source*: Adapted from Sweeney et al. [153]

The BBB plays a critical role in protecting the brain and facilitating the transport of substances. There are two main pathways for substances to cross the BBB: transcellular transport, where molecules move through endothelial cells from the luminal to the abluminal surface and into the brain parenchyma, and paracellular transport, which occurs through tight junctions between endothelial cells. Transcellular transport is influenced by factors such as concentration, electrical charge, and lipophilicity, allowing substances to traverse based on their electrochemical gradient. However, two key mechanisms, particularly relevant for drug delivery, are transmembrane diffusion and transporter-mediated processes . The integrity of the BBB plays a pivotal role in maintaining the neurovascular unit’s (NVU) function and supporting the brain’s intricate network (Fig. 1). The NVU, composed of endothelial cells, pericytes, astrocytes, neurons, and extracellular matrix components, plays a central role in maintaining BBB integrity and regulating substance transport into the brain. Astrocytes especially provide structural and metabolic support, as well as regulating the barrier’s permeability. Pericytes further contribute by stabilizing endothelial cells and influencing BBB integrity. In addition to these cellular components, the BBB interacts with neurons, microglia, and extracellular matrix proteins, forming a dynamic and complex interface essential for the brain’s protection and homeostasis. Together, these elements ensure a tightly regulated environment, necessary for proper neuronal function and protection from systemic fluctuations or harmful agents (Fig. 1).

## Strategies for improved delivery of therapeutics to the brain

Drug delivery approaches encompass a wide range of techniques, including the use of microspheres, biodegradable wafers, and various colloidal carrier systems. These systems include liposomes, nanoparticles, nanogels, dendrimers, micelles, nanoemulsions, polymersomes, exosomes, and quantum dots, each offering unique properties for enhancing therapeutic efficacy and TD [1]. Convection-enhanced delivery enables the direct injection of therapeutics, including gene therapies, into the brain, allowing for widespread distribution [36]. Also, non-viral nanocomplexes are being studied as vectors for brain gene therapy, aiming to optimize delivery and reduce the need for multiple injections [158]. Moreover, small peptides under 500 Da can diffuse across the BBB passively, but low penetration rates hinder their effectiveness. Enhancing lipophilicity or hydrogen bonding can improve brain absorption but may also increase peripheral uptake [122]. Biologics, such as monoclonal antibodies, peptides, and NA-BTs, face significant challenges in crossing the BBB due to their large and complex nature, which limits brain absorption compared to smaller drugs. Unlike small lipophilic drugs, biologics lack natural transport mechanisms to facilitate BBB passage and cannot rely on systemic circulation alone for brain penetration. This limitation calls for alternative methods, such as receptor-mediated transport or invasive delivery systems. To address these barriers, strategies are being developed to engineer biologics for enhanced BBB permeability and create advanced delivery platforms, such as nanoparticles and exosomes, which show promise for improving biologic-based therapies in the CNS .

## Targeting BBB

One approach to enhance medication delivery to the brain is to induce controlled disruptions in the BBB, similar to what occurs in various inflammatory CNS diseases. For instance, studies have shown that astrocytic expression of vascular endothelial growth factor A (VEGF-A) plays a crucial role in regulating BBB permeability in a mouse model of MS [13]. This finding highlights the significant impact that VEGF-A, secreted by astrocytes, has on the integrity of the BBB, contributing to its dysfunction and facilitating the infiltration of immune cells into the brain during MS. Moreover, small compounds and allosteric regulators can potentially improve drug delivery to the brain by modifying BBB transporter activity. While enhancers and inhibitors can control this activity, most BBB transporter structures and their allosteric binding sites are still unidentified. An α-adrenergic receptor agonist, for instance, has been used to improve the BBB penetration of anorectic leptin [10]. Recent studies have shown that claudin-5 interaction inhibitors cause a reduction in claudin-5 levels on the cell membrane by promoting internalization and downregulation. This process explains how claudin-5 interaction inhibitors can reduce the functionality of claudin-5, thereby increasing BBB permeability [21].

### Targeting transporters

To target BBB transporters, researchers can design analogs of endogenous Ligands that effectively bind to both the transporter and a receptor in the CNS. These analogs are structurally and Functionally similar to natural Ligands, allowing them to serve as ligands for the BBB transporter and the CNS receptor. Transporter-based delivery systems, such as those utilizing the L-type amino acid transporter 1 (LAT1), offer a promising approach for delivering therapeutic agents across the BBB. LAT1 facilitates the uptake of large neutral amino acids like leucine and phenylalanine into the brain, and therapeutic compounds can be conjugated to LAT1 substrates to exploit this pathway for drug delivery [61]. However, this strategy has several limitations. Substrate competition can occur, as endogenous amino acids may outcompete drug conjugates for transporter binding, reducing delivery efficiency. Additionally, limited transport capacity may restrict the amount of therapeutic agent that can be delivered. Transporter expression can also vary between individuals and across disease states, leading to inconsistent drug uptake. These factors collectively pose challenges in achieving reliable, targeted, and effective delivery via transporter-mediated mechanisms.

Additionally, targeted drug delivery to the brain can be facilitated by designing specific peptides or small-molecule analogs that selectively bind to various BBB transporters, such as peptide transporter 1 (PEPT1), monocarboxylate transporter 1 (MCT1), and organic anion transporting polypeptides (OATPs). These transporters, which normally mediate the uptake of essential nutrients and metabolites into the brain, can be exploited to carry therapeutic agents across the BBB. By mimicking the natural substrates of these transporters, drugs can effectively “hitchhike” across the barrier, improving their brain bioavailability [131]. This strategy enhances CNS-targeted drug delivery but also requires careful consideration of transporter specificity, substrate competition, and variability in expression levels across individuals and disease states.

Furthermore, transferrin receptors (TfRs) expressed on the BBB play a key role in mediating iron uptake into the brain. This physiological pathway can be harnessed for drug delivery by conjugating therapeutic agents or nanoparticles with transferrin, the receptor’s natural ligand, to enable receptor-mediated transcytosis. This active transport mechanism allows the drug-conjugates to bind to TfRs, undergo internalization, and be transported across the endothelial cells into the brain parenchyma, thereby enhancing drug penetration into the CNS. This approach has shown promise in improving the delivery efficiency of biologics and nanomedicines targeted to neurological disorders [69].

Active transport is an energy-dependent process that utilizes ATP to move substances against their concentration gradients. This mechanism encompasses several pathways, including the activity of pericytes and endothelial ion transporters, solute carrier proteins, and ATP-binding cassette (ABC) transporters involved in active efflux [153]. Some transporters at the BBB function as efflux pumps, actively removing substances from the brain back into the bloodstream. Inhibiting these efflux transporters could enhance drug penetration through the BBB and increase their retention within the CNS, particularly for drugs that struggle to accumulate in the brain. To effectively inhibit efflux, it is essential to identify the specific transporters involved and target them for inhibition. One well-known efflux transporter is P-glycoprotein (P-gp), which limits the effectiveness of many therapeutic drugs [30]. By blocking the action of efflux transporters, drugs can remain in the brain for longer periods, thus enhancing their therapeutic impact. Efflux mechanisms often involve multiple efflux systems, and efflux transporters typically have a broad range of ligands. As a result, inhibiting efflux transporters can affect the distribution of various medications and endogenous substances [24].

### Targeting cells

The components of the NVU form a complex network that works together to sustain the homeostatic microenvironment essential for neuronal function [165, 168]. Current therapeutic strategies primarily focus on neuronal signaling pathways while largely overlooking the crucial NVU mechanisms, which may be a contributing factor to the limited effectiveness of existing treatments. Enhancing the function of the NVU could improve neuronal survival and provide more efficient therapies for CNS disorders [169]. One potential approach is to target interactions between BBB endothelial cells and immune cells, which could help block immune cell infiltration into the brain. For instance, natalizumab is a monoclonal antibody that selectively targets α4-integrin on immune cells, thereby limiting their infiltration into the CNS in MS patients [31]. In parallel, an emerging approach involves brain-targeted cell-membrane cloaking, in which nanoparticles are enveloped with membranes derived from brain-tropic cells such as microglia or neurons. This biomimetic strategy enhances nanoparticle transport across the BBB by leveraging natural cellular trafficking mechanisms while concurrently minimizing immune recognition and clearance. In this context, researchers have recently developed cancer cell membrane-cloaked biomimetic nanoparticles for the TD of Signal Transducer and Activator of Transcription 3 (STAT3) siRNA to glioblastoma (GBM). By cloaking nanoparticles with membranes derived from homologous cancer cells, these systems exploit the natural homotypic binding properties of tumor cells, enabling enhanced recognition and uptake by GBM cells [89].

Engineered immune-exosomes, particularly those modified to express surface ligands for endothelial or neuronal receptors, also exploit NVU components for precise delivery of drugs [163]. Within the NVU, pericytes have gained attention not only for their role in regulating BBB permeability but also for their potential as therapeutic targets. Pericyte dysfunction is associated with BBB breakdown in various neurodegenerative diseases, and therapies aimed at stabilizing or modulating pericyte function can improve barrier integrity and influence the transcytosis and paracellular transport pathways [151, 187]. Maintaining pericyte function is also essential for preserving BBB integrity in a range of diseases. The loss of pericytes disrupts the BBB, necessitating the development of treatments that can cross this barrier. For instance, metabolic carbonic anhydrase inhibitors have been shown to protect pericyte function in conditions such as diabetes [134]. However, challenges remain in precisely targeting specific cells, loading drug payloads, and ensuring their effective release within the CNS. Consequently, these interventions can significantly alter the delivery kinetics of nanocarriers and NA-BTs by enhancing their retention, distribution, and uptake in the brain microenvironment, highlighting the NVU’s expanding role in next-generation CNS drug delivery systems.

### Pharmacokinetic and pharmacodynamic considerations

Developing systems for transporter-mediated brain delivery is challenging due to the brain’s unique physiology and the presence of various barriers, which complicates the assessment of delivery success. To evaluate the effectiveness of transporter-utilizing (pro)drugs and nanocarriers, it is essential to integrate CNS pharmacokinetics (PK), which accounts for the absorption, distribution, metabolism, and excretion of drugs within the brain [48]. A critical component of understanding a drug’s pharmacodynamic (PD) response is accurately estimating its concentration at the target site in the brain. Furthermore, absorption, distribution, metabolism, and excretion processes play significant roles in determining bioavailability and should be carefully considered [59]. There is also a risk that prodrugs or nanocarriers may unintentionally transfer the parent drug to non-target tissues, which could lead to unintended side effects or toxicity. Therefore, the optimal strategy is to use transporters to direct (pro)drugs to the brain and release the active drug specifically at the target site. Efficacy should be evaluated based on BBB permeability, brain delivery, and intra-brain distribution [58]. Additionally, pharmacokinetic studies are necessary to confirm the effectiveness of nanocarriers in achieving targeted brain delivery.

## Nanoparticle delivery systems in the brain

**Fig. 2:**
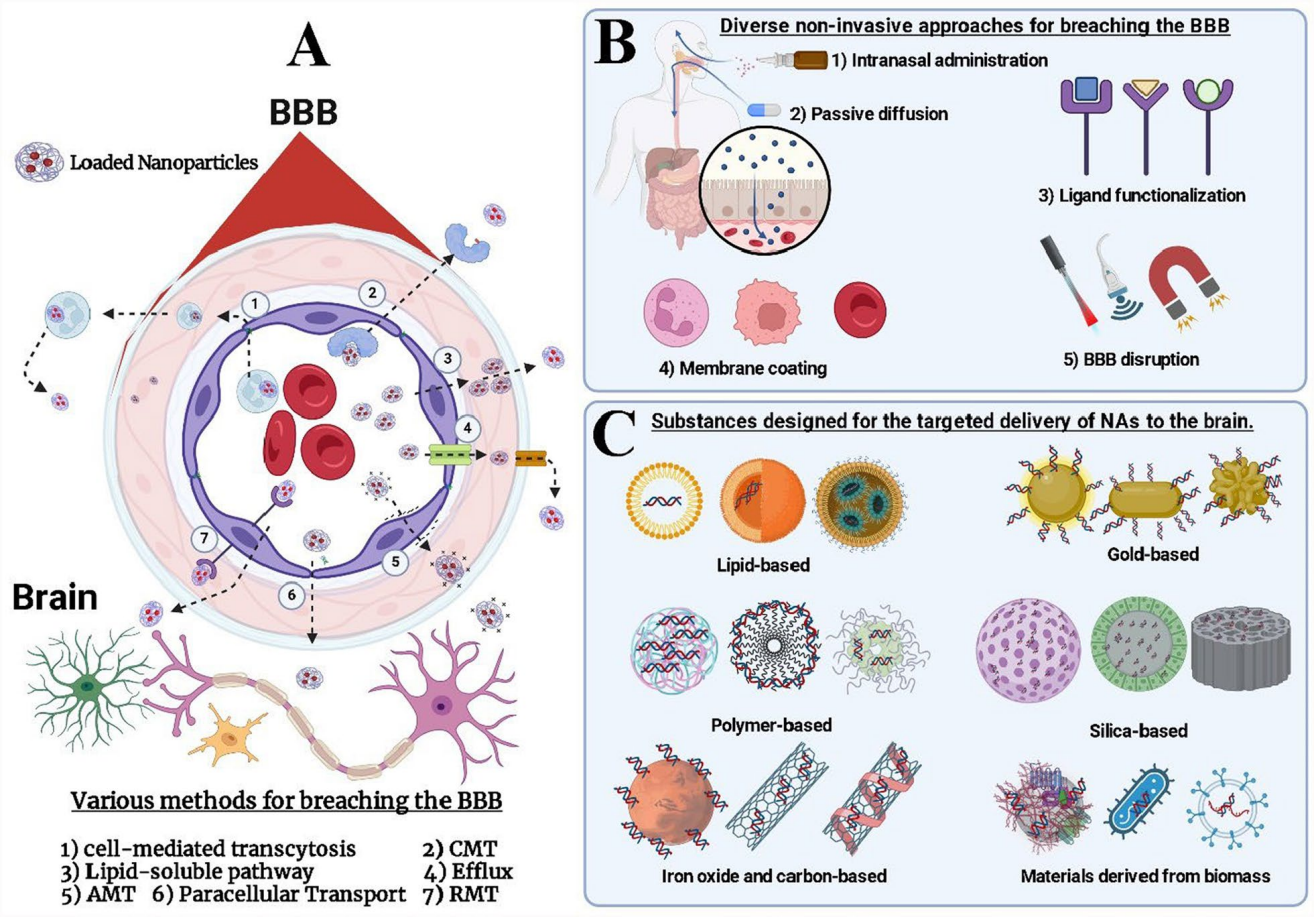
Summary of nanoparticle-based systems, non-invasive approaches, and targeted delivery (TD) in the brain. **A** The image illustrates seven key methods for overcoming the blood–brain barrier (BBB): Cell-mediated transcytosis: Immune or stem cells carrying drug payloads traverse the BBB. Carrier-Mediated Transport (CMT): Drugs mimic substrates of transporters like GLUT1 or LAT1 to gain entry. Lipid-Soluble Pathway: Lipophilic drugs diffuse passively through the lipid-rich BBB. Efflux Mechanisms: Strategies to bypass efflux transporters like P-gp, which expel drugs from the brain. Adsorptive-Mediated Transcytosis (AMT): Positively charged carriers interact with endothelial cell surfaces for transport. Paracellular Transport: Temporary BBB disruption enables substances to pass between endothelial cells. Receptor-Mediated Transcytosis (RMT): Drugs conjugated to ligands target receptors like transferrin or low-density lipoprotein receptor (LDLR) to cross the BBB. The titles of parts **B** and **C** are demonstrated in the picture. *Source*: Adapted from Wu et al. [173]

Nanoparticles (NPs) exhibit unique properties that enable them to interact effectively with biological systems, offering significant advancements in drug delivery, particularly to the CNS. Their ability to cross the BBB has positioned them as a promising tool for treating neurological disorders. Despite progress in nanoparticle-based drug delivery, significant challenges persist. One major obstacle is the action of efflux transporters like P-gp at the BBB, which can actively remove drug-loaded nanoparticles even after they have crossed the barrier. This efflux activity substantially reduces the therapeutic effectiveness of such delivery systems [188]. The advancements in incorporating materials and surface modifications allowed NPs to bypass efflux mechanisms or utilize receptor-mediated pathways for more effective delivery [104]. Some nanoparticle designs exploit adsorptive-mediated transcytosis (AMT), a process that relies on electrostatic interactions between positively charged nanoparticles and the negatively charged luminal surface of endothelial cells at the BBB. This non-specific uptake enhances transcytosis efficiency but may lack the targeting precision of receptor-based systems. In contrast, receptor-mediated transcytosis (RMT) is a highly specific and efficient mechanism for transporting therapeutic agents-including nanoparticle-based formulations-across the BBB. In this approach, nanoparticles are functionalized with ligands such as peptides, antibodies, or aptamers that selectively bind to receptors abundantly expressed on BBB endothelial cells (e.g., transferrin receptor, insulin receptor, or low-density lipoprotein receptor). Upon ligand–receptor binding, the complex undergoes endocytosis, is trafficked across the cell via endosomal pathways, and is subsequently released on the abluminal side, enabling precise delivery of the therapeutic payload into the brain. Combining AMT and RMT strategies, or engineering multi-functional nanoparticles, can further enhance BBB permeability and targeting efficiency for neurological therapies. Additionally, hybrid nanoparticles combine the strengths of multiple delivery systems, such as liposomal and polymeric components, to achieve enhanced stability, controlled drug release, and TD [8]. These approaches not only improve BBB penetration but also ensure effective distribution within the brain, minimizing off-target effects and enhancing therapeutic outcomes (Fig. 2).

### Liposomal nanoparticles

Liposomes, spherical lipid-based carriers, are widely utilized for drug delivery but face challenges such as rapid plasma clearance by immune macrophages, limiting their circulation time [63]. To overcome this, modifications like grafting with gangliosides or polyethylene glycol (PEG) have been developed. PEG provides steric stabilization and protection, extending the half-life of liposomes. TD has shown promise, as demonstrated in preclinical trials where liposomes conjugated with the anti-Transferrin R antibody successfully crossed the BBB via transferrin receptor-mediated endocytosis [68]. Moreover, peptide-grafted liposomes have enhanced brain drug delivery, significantly increased drug uptake, and reduced glioma viability [40]. Innovations such as incorporating viral proteins with liposomes have further improved the delivery of neuroprotective agents to the CNS [62]. Additionally, liposomes can self-assemble with nucleic acids like DNA or siRNA, forming multi-lamellar lipoplexes. These modified liposomes hold significant potential for enhancing CNS drug delivery by combining TD, prolonged circulation, and advanced surface modifications.

### Nanostructured lipid carriers

Nanostructured lipid carriers (NLCs) are advanced drug delivery systems composed of biodegradable solid and liquid lipids. Their unique, less-ordered crystalline structure minimizes drug leakage and enables higher drug loading capacity. NLCs are highly effective in reducing the toxicity of chemotherapeutic drugs and enhancing brain drug delivery by overcoming efflux mechanisms at the BBB. Their attributes, including high biocompatibility, controlled drug release, and efficient encapsulation, make them ideal for bypassing or crossing the BBB with minimal toxicity [25]. In antiretroviral therapy, NLCs have significantly improved brain delivery of drugs such as indinavir, achieving a remarkable 400-fold increase in CNS uptake [73]. They also enhance BBB permeability for lipophilic compounds like tanshinone-1 and have been adapted for various administration routes [174]. Furthermore, NLCs have shown therapeutic efficacy in treating conditions like MS, as evidenced by their successful application in preclinical mouse models [44]. This versatility and effectiveness position NLCs as a promising platform for delivering a range of therapeutics to the brain, addressing challenges in CNS drug delivery.

### Polymeric nanoparticles

Polymeric nanoparticles (PNPs) provide a versatile platform for drug delivery, leveraging diverse monomers and polymerization techniques for customization. These nanoparticles, including synthetic, natural, and hybrid varieties, are particularly effective for brain targeting due to their small size, tunable properties, and beneficial physical characteristics. PNPs enable controlled drug release, protect therapeutic agents from degradation, and improve bioavailability [126]. Polyplexes, formed by combining positively charged polymers with negatively charged DNA or siRNA, expand the utility of PNPs for delivering genetic material [57]. PNPs are promising tools for enhancing drug penetration across the BBB. For example, poly(lactide-co-glycolide) acid (PLGA) nanoparticles have been used to encapsulate doxorubicin and siRNA, showing efficacy in glioma treatment by enhancing drug delivery across the BBB [107]. Additionally, receptor-mediated transport can be enhanced by modifying PNPs to target specific receptors. For instance, antibody-grafted chitosan nanoparticles loaded with siRNA have demonstrated the ability to halt HIV replication in the brain, highlighting their potential for treating CNS diseases [52]. The flexibility in design, efficient drug delivery, and ability to cross the BBB make PNPs a promising strategy for addressing challenges in CNS drug therapy and targeting neurological disorders effectively.

### Peptide-derivatives nanocarriers

Short peptides, typically 5–30 amino acids long, have gained recognition as efficient carriers for delivering nucleic acids both in vitro and in vivo. These peptide-based carriers effectively address the challenges associated with nucleic acid transport, proving particularly adept at delivering DNA and siRNA [50]. Peptides used for this purpose are classified into two main categories: cell-penetrating peptides (CPPs) and homing peptides (HPs). CPPs are short, cationic sequences capable of penetrating cell membranes via receptor-independent mechanisms. In contrast, HPs are selected using phage display techniques and utilize receptor-mediated endocytosis to achieve TD. Both types of peptides enable NAD through mechanisms such as PNA coupling and co-self-assembly. These processes give rise to various nano-vehicles, including nanoparticles, nanofibers, and nanotubes [164]. Recent advancements have demonstrated the potential of peptide modifications for enhanced delivery efficiency. For example, modifying the rabies virus glycoprotein (RVG) with additional arginine residues to create RVG-9R significantly improved siRNA delivery across the BBB and into the brain [161]. The versatility and efficiency of these short peptides make them a promising tool for advancing NAD technologies, particularly for applications targeting the CNS and beyond.

### Inorganic nanostructured molecules

Inorganic nanoparticles, including gold, silver, and silica, are increasingly utilized for CNS drug delivery. Gold nanoparticles (AuNPs) efficiently penetrate the BBB and exhibit low toxicity, with exosome-coated AuNPs enhancing brain cell binding and transport [76]. Silver nanoparticles (AgNPs) show potential for binding serotonin and enhancing chemotherapeutic effects on GBM, but their toxicity needs further investigation [92]. Silica nanoparticles, often functionalized with PEG and combined with lactoferrin, enhance BBB penetration through receptor-mediated endocytosis, making them versatile for CNS delivery [154]. Other transition metal nanoparticles, like selenium [60] and superparamagnetic iron oxide [49], enhance CNS delivery, with selenium showing promise in Alzheimer’s by interacting with amyloid-beta plaques and improving memory. Recently, pH-sensitive drug delivery systems like superparamagnetic iron oxide nanoparticles loaded with doxorubicin optimize drug release in GBM treatment [49]. Moreover, Carbon dots, known for biocompatibility and fluorescence, enhance pDNA and siRNA delivery to the CNS [114].

## Extracellular vesicles as novel drug delivery systems

Exosomes are rapidly emerging as one of the most promising innovations in drug delivery. These naturally occurring nanocarriers have evolved to transport complex biological molecules across challenging barriers in the body, like the BBB, with remarkable precision and minimal immune response. Their natural compatibility with the human body, ability to remain stable in circulation, and potential for TD make them a compelling solution to many of the limitations faced by current therapeutic delivery systems in the brain [17, 27].

Their nanoscale size (30–150 nm) and endogenous origin facilitate efficient cellular uptake and systemic circulation while minimizing immune clearance. Moreover, surface proteins such as tetraspanins and integrins enable exosomes to exhibit cell-specific targeting capabilities . Recent developments in engineering strategies, including sonication, electroporation, and chemical surface modifications, have expanded the utility of exosomes for loading a broad spectrum of therapeutic cargos such as nucleic acids, proteins, and small molecules [26]. In particular, stem cell-derived exosomes have demonstrated immunomodulatory and regenerative properties, while milk-derived exosomes have shown promise for oral delivery of hydrophilic biomolecules, enhancing bioavailability and transepithelial transport [91, 156]. Despite their therapeutic potential, challenges such as large-scale production, standardization of isolation techniques, and batch-to-batch reproducibility remain critical hurdles for clinical translation [165, 168]. Nevertheless, ongoing preclinical and clinical studies underscore the value of exosomes as next-generation delivery platforms for diverse therapeutic modalities in oncology, neurology, infectious diseases, and regenerative medicine [130].

EVs facilitate intercellular communication in the brain and have the potential to cross the BBB, offering advantages over traditional drug delivery systems by targeting specific cells, maintaining stability in the bloodstream, and delivering therapeutic materials. Current research is exploring the bioengineering of EVs for stroke therapy, their interactions with the BBB, and the challenges and prospects of EV-based therapies for brain disorders [5, 18]. A study by Morad et al. [115] investigated how breast cancer-derived EVs cross the intact BBB, revealing transcytosis as the primary mechanism and outlining the cellular pathways involved. This finding highlights the potential for EVs as new methods for drug delivery in treating brain disorders. MSCs and their secreted EVs have shown promise in promoting neurological recovery after ischemic events. These EVs contribute to brain remodeling, immune modulation, and enhance angiogenesis and neurogenesis [120]. Additionally, neural cells, including microglia, oligodendrocytes, astrocytes, and neurons, rely on EVs for essential intercellular communication, which influences neural specialization, cell growth, and synapse formation [3]. In the context of CNS disorders, EVs are also gaining attention as potential vehicles for delivering therapeutic agents to the brain. Their ability to address currently untreatable CNS conditions opens up new avenues for treatment [138]. Exosomes, a subtype of EVs, are emerging as powerful tools in nanotechnology for drug delivery. They offer high biocompatibility, efficient drug delivery, and the ability to cross physiological barriers with minimal side effects, making them an exciting area of focus for therapeutic interventions [128].

### Exosome biogenesis and its biodistribution

**Fig. 3:**
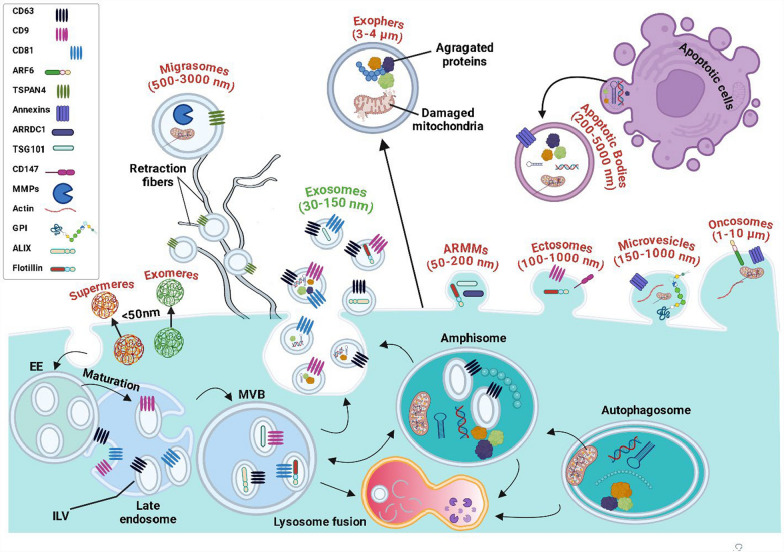
Overview of different types of extracellular vesicles (EVs). The illustration presents a comprehensive overview of various EVs, emphasizing their structural and functional diversity. Each EV type varies in size, origin, and cargo, offering unique capabilities for therapeutic applications and intercellular communication. Key EV types depicted include: Supermeres and Exomeres: Nanoparticles without lipid bilayer, notable for their unique biomolecular cargo and functions in intercellular communication, metabolic, and signaling processes. Migrasomes: Large vesicles formed during cell migration, playing roles in intercellular signaling and cargo transport. Exosomes: Well-characterized small vesicles derived from the endosomal pathway, crucial for transporting proteins, lipids, and nucleic acids across cells. ARMMs (Arrestin Domain-Containing Protein 1-Mediated Microvesicles): Specialized small vesicles involved in signaling pathways, formed through plasma membrane budding. Ectosomes: A subset of small vesicles with specific size ranges and functional properties. Microvesicles: Larger vesicles formed by direct outward budding of the plasma membrane, involved in cell-to-cell communication and immune modulation. Oncosomes: Large vesicles released from cancer cells, associated with tumor progression and metastasis. Exophers: are formed when cells expel large portions of their cytoplasm, along with cellular components such as organelles, aggregated proteins, and other debris. *Source*: Adapted from Jeppesen et al. [66]

Exosomes, a subtype of extracellular vesicles, are emerging as a promising tool for brain drug delivery, especially in the context of NA-BTs. Exosome biogenesis is intricately linked to the endosomal trafficking pathway, beginning with the invagination of the plasma membrane, which forms early endosomes. These early endosomes undergo maturation into late endosomes or multivesicular bodies (MVBs). A hallmark feature of MVBs is the formation of intraluminal vesicles (ILVs) within their lumen, generated by a secondary inward budding of the endosomal limiting membrane. This budding process sequesters specific cytosolic components, including proteins, lipids, DNA fragments, mRNAs, microRNAs (miRNAs), long non-coding RNAs (lncRNAs), and other bioactive molecules, into the ILVs (Fig. 3). The formation of ILVs involves both ESCRT-dependent and ESCRT-independent mechanisms. In the ESCRT (Endosomal Sorting Complex Required for Transport) pathway, the process is orchestrated by four complexes-ESCRT-0, I, II, and III- along with associated proteins like ALG-2-interacting protein X and Tumor Susceptibility Gene 101, which help recognize ubiquitinated cargo, deform the membrane, and facilitate vesicle scission [70]. In parallel, ESCRT-independent mechanisms involve molecules like tetraspanins (e.g., CD9, CD63, CD81) and lipid components such as ceramides, which can drive vesicle budding through changes in membrane curvature and fluidity [65]. Once ILVs are formed within MVBs, these MVBs have two potential fates: fusion with lysosomes for degradation or fusion with the plasma membrane, resulting in the release of ILVs into the extracellular space. At this stage, ILVs are termed exosomes, now functioning as intercellular messengers capable of delivering their cargo to recipient cells via endocytosis, phagocytosis, or direct membrane fusion [55]. This complex and highly regulated process enables exosomes to play a central role in cell-to-cell communication and offers a sophisticated platform for therapeutic delivery. Exosomes are categorized based on their cellular origin, biogenesis pathways, and surface markers, which are crucial for developing effective delivery strategies [71] (Fig. 3). The identification and characterization of exosomes typically involve the use of surface markers such as tetraspanins and heat shock proteins (e.g., HSP70, HSP90) [190], which help define their cellular origin and functional properties. Exosomes are also classified based on their formation pathways, which include the ESCRT pathway, as well as ESCRT-independent mechanisms [55]. The main sources of exosomes for brain delivery include MSCs, endothelial cells, dendritic cells, and neural cells. Understanding the classifications and biodistribution of these exosomes is essential for optimizing their use in targeted therapies. MSC-derived exosomes are highly valued for their ability to modulate the immune system, which is vital for managing neuroinflammation, offering neuroprotection, and supporting tissue regeneration in neurological disorders [147]. Neural cells-derived exosomes naturally possess the ability to target specific brain regions, making them an excellent option for precise delivery to diseased or injured areas, thereby promoting neural repair [192]. Furthermore, exosomes originating from endothelial cells can interact directly with the BBB, enhancing their specificity in transporting therapeutic agents to the CNS [150]. Moreover, exosomes derived from dendritic cells have demonstrated significant potential in modulating immune responses, particularly by enhancing antigen presentation and regulating immune tolerance. These immunomodulatory properties make them promising candidates for the development of therapeutic vaccines aimed at brain tumors and neurodegenerative disorders [177].

### Exosomes as nucleic acid delivery (NAD) machinery

The distribution of exosomes is significantly influenced by their size, surface characteristics, and targeting strategies. When administered systemically, exosomes can cross the BBB and accumulate in brain tissues [135]. Different cell types-derived exosomes exhibit varying affinities for the brain. To further enhance targeting accuracy, exosomes can be engineered by modifying their surface with ligands, antibodies, or peptides, enabling them to specifically target certain brain cells or regions [43]. Exosomes are increasingly being recognized as effective carriers for transporting nucleic acids to the brain [15]. Therapeutic nucleic acids (such as siRNA, miRNA, mRNA, and antisense oligonucleotides) can be integrated into exosomes using either endogenous or exogenous loading strategies, each leveraging distinct molecular mechanisms. In endogenous loading, nucleic acids are introduced into the exosome-producing cells before exosome release, typically via plasmid transfection, synthetic RNA delivery, or viral vector infection. These nucleic acids are synthesized intracellularly and selectively incorporated into ILVs during their formation within MVBs. This selective packaging is mediated by specific RNA-binding proteins (RBPs) such as heterogeneous nuclear ribonucleoprotein A2/B1, Y-box binding protein 1, and SYNCRIP, which recognize particular sequence motifs or secondary structures on target RNAs and actively guide them into the exosomal cargo. While this strategy ensures a more physiological context and compatibility with the exosome’s endogenous biogenesis pathways, the efficiency of targeted loading is variable and dependent on the precise sorting signals and expression levels [127]. In contrast, exogenous loading involves the post-isolation introduction of nucleic acids into purified exosomes. Among the most commonly used techniques is electroporation, which uses controlled electrical pulses to transiently permeabilize the exosomal lipid bilayer, allowing nucleic acids to enter the vesicle lumen; however, this method may cause cargo aggregation or compromise membrane integrity if not finely tuned. Another approach is chemical transfection, which employs cationic lipids or polymers to form nucleic acid complexes that can fuse with or be endocytosed by exosomes. While this method is gentler on exosomal structure, it may introduce potentially cytotoxic residues. Lastly, passive incubation entails the co-incubation of exosomes with nucleic acids under conditions that favor spontaneous membrane association or diffusion. Though this technique preserves vesicle stability, it typically results in lower loading efficiency and lacks cargo specificity. Together, these strategies offer a versatile toolkit for engineering exosome-based delivery systems, each with trade-offs between precision, efficiency, and biocompatibility [99]. Recent clinical studies have underscored the potential of using exosomes in nucleotide-based gene therapy. For example, a study showed that exosomes loaded with miR-146b, derived from bone marrow stromal cells (BMSC), significantly reduced tumor metastasis by approximately 60% [74]. These findings highlight the growing potential of exosome-based therapies in treating neurological conditions and cancers through precise NAD.

## The role of exosomes in CNS disorders

Exosomes have emerged as a highly promising tool for treating brain diseases due to their exceptional biocompatibility and unique ability to penetrate the BBB. Their natural role in intercellular communication and cargo delivery positions them as effective carriers for therapeutic agents. Recent research highlights their potential application in NDDs, particularly for regulating synaptic function and restoring neuronal health [101, 102]. For instance, a notable study demonstrated that MSCs-derived exosomes, when loaded with gold nanoparticles, were able to selectively target inflamed brain regions hallmark of many NDDs [123]. This selective targeting underscores their potential to deliver therapeutic agents precisely to areas of the brain affected by disease, minimizing off-target effects and enhancing treatment efficacy. In this section, we discuss recent advancements in understanding the role of exosomes in neurodegenerative diseases, highlighting their potential as both biomarkers and therapeutic delivery systems.

### Exosomes in Alzheimer’s disease

Alzheimer’s disease (AD) is an important NDD, primarily characterized by the progressive decline in memory and cognitive functions, severely affecting the quality of life of millions worldwide [141]. Emerging research has identified EVs, particularly exosomes, as critical mediators in both the progression and potential treatment of AD [132, 184]. These nanoscale vesicles can transport pathogenic molecules, such as amyloid-beta (Aβ) peptides and tau proteins, facilitating the spread of disease pathology. However, their unique properties also make them attractive candidates for therapeutic applications. More than a decade ago, Alvarez-Erviti et al. [9] introduced a groundbreaking strategy for treating neurological disorders by utilizing engineered exosomes derived from dendritic cells, demonstrating their potential to deliver siRNA across the BBB. While this study marked a significant advancement in exosome-mediated brain delivery, subsequent research has shown that the source of exosomes plays a crucial role in their biodistribution and therapeutic efficacy. Dendritic cell-derived exosomes, as used in the Alvarez-Erviti study, are well-suited for immunomodulatory applications and can be engineered for TD. However, MSC-derived exosomes have gained greater popularity due to their inherent regenerative and anti-inflammatory properties, as well as their ability to home to sites of injury or inflammation, including the CNS. Comparative studies suggest that while both exosome types can be modified for brain-TD, MSC-derived exosomes may offer advantages in terms of safety profile, scalability, and intrinsic therapeutic potential, making them highly attractive for clinical applications in brain injury [100, 144]. Moreover, hippocampal neuronal stem cell (NSC)-derived exosomes loaded with specific microRNAs (miR-322, miR-17, miR-485) have shown remarkable efficacy in preclinical studies. These exosomes improved memory deficits and enhanced long-term potentiation by mitigating the neurotoxic effects of Aβ oligomers, suggesting a promising avenue for restoring cognitive functions in AD patients [110].

### Exosomes in Parkinson’s disease

Parkinson’s disease (PD) is the second most common NDD, characterized by the progressive degeneration of dopamine-producing neurons in the substantia nigra, leading to diminished dopaminergic activity and motor impairments [12]. A hallmark of PD pathology is the presence of Lewy bodies, which consist primarily of aggregated α-synuclein (α-Syn), along with neuroinflammation, microglial activation, and oxidative stress in the brain [155]. It has been demonstrated that neuronal dysfunction and gene modulation mediated by non-coding RNAs play a significant role in Parkinson's disease and other synucleinopathies. These molecules may also serve as promising candidates for new therapeutic approaches [109]. Advances in exosome-based therapies have opened new possibilities for delivering therapeutic agents to the brain. For instance, Yang et al. utilized MSC-exosomes to deliver ASOs to the brains of A53T transgenic PD mice. This innovative approach demonstrated the potential to reduce α-Syn levels and alleviate PD symptoms [178]. In addition to targeting α-Syn, exosomes have been explored for mitigating oxidative stress, another key contributor to PD pathology. Serum-derived exosomes loaded with miR-137 effectively reduced oxidative stress in neurons, leading to physiological and behavioral improvements in PD animal models [67].

### Exosomes in amyotrophic lateral sclerosis

Amyotrophic lateral sclerosis (ALS) is a devastating NDD characterized by the progressive degeneration of motor neurons in the motor cortex, brainstem, and spinal cord, ultimately leading to muscle paralysis and death [29, 64]. Despite significant advancements in understanding its pathology, effective treatments remain limited. Recent research has highlighted the potential of exosome-based therapies as promising candidates for ALS treatment due to their neuroprotective properties and ability to target multiple aspects of the disease. Adipose-derived stem cells (ADSCs)-derived exosomes have shown considerable neuroprotective effects in ALS models. These exosomes were found to enhance the survival of motor neuron-like NSC-34 cells by increasing the expression of human SOD1, a critical antioxidant enzyme, and activating anti-apoptotic pathways [20]. Further proteomic analyses identified 189 proteins within these exosomes that play roles in cell adhesion and apoptosis prevention, including the downregulation of the pro-apoptotic gene *Bax* and the upregulation of the anti-apoptotic gene Bcl-2 [19]. Mitochondrial dysfunction, a hallmark of ALS pathology, has also been targeted using exosome-based approaches. ADSC-derived exosomes have demonstrated the ability to restore mitochondrial function, reduce the accumulation of mutant SOD1 proteins, and enhance mitochondrial coupling efficiency in ALS models [22, 85]. Specifically, these exosomes improved mitochondrial membrane potential and complex I activity in mutant SOD1 (G93A) NSC-34 cells, indicating their potential to mitigate mitochondrial impairments associated with ALS [22].

### Exosomes in Huntington’s disease

Huntington’s disease (HD) is a progressive NDD characterized by the expansion of CAG repeats in the huntingtin gene, resulting in a range of symptoms, including neuropsychiatric disturbances, cognitive decline, and involuntary movements [83]. Although the genetic mutation responsible for HD is well-defined, the exact molecular mechanisms driving the disease are still not fully understood. Recent research has focused on developing various therapeutic approaches to address this gap, with a particular emphasis on targeting the RE1-silencing transcription factor (REST) as a potential treatment in HD and other neurodegenerating diseases [14]. One promising study by Lee et al 86. utilized exosomes to deliver miR-124, a small RNA molecule, to R6/2 HD mice, aiming to restore REST function. These exosomes were engineered from HEK293T cells transfected with the pSUPER-mir-124 vector and successfully reduced REST expression. Despite this, behavioral improvements were not observed in the treated mice, suggesting that further refinement of this therapeutic strategy is needed. Moreover, gene-editing technologies like prime editors have emerged as promising tools for directly correcting the genetic mutations that cause Huntington’s disease, potentially offering a more precise approach to targeting the root cause [86]. As research advances, these methods, together with RNA-based therapies and neuroprotective agents, hold significant potential to improve future treatments for HD.

### Exosomes in epilepsy

Epilepsy is a neurological condition marked by abnormal electrical activity in the brain, which leads to seizures, altered sensations, and episodes of impaired consciousness. It is frequently associated with several NDDs, and current pharmacological treatments often provide only partial symptom relief, with limited long-term effectiveness and poor overall prognosis [23]. Given the challenges of managing epilepsy, alternative therapeutic strategies are being explored. One study highlighted specific exosomal proteins, such as F9 and TSP-1, present in the serum of individuals with epilepsy, suggesting their potential as biomarkers for the detection of epilepsy [96]. In another study, exosomes loaded with miR129-5p demonstrated protective effects on neurons by reducing degeneration caused by status epilepticus, achieved through the inhibition of the pro-inflammatory HMGB1/TLR4 signaling pathway [101]. Additionally, research on tuberous sclerosis complex, a genetic disorder commonly linked to epilepsy, found that exosomes released from epileptogenic TSC tubers served as carriers of pro-inflammatory microRNAs [35].

### Exosomes in stroke

Stroke, resulting from inadequate blood supply to the brain, can lead to significant disability or even death. Despite considerable research efforts, over 700 drugs aimed at improving stroke outcomes have failed to achieve clinical approval [170]. In recent years, exosomes have emerged as a promising therapeutic approach for enhancing recovery following stroke. These extracellular vesicles, naturally secreted by various cells, have demonstrated the potential to promote neuroprotection and support tissue regeneration after brain injury [7]. For example, MSCs-derived exosomes that overexpress miR-133b have demonstrated neuroprotective effects by enhancing neuroplasticity and promoting functional recovery post-stroke [176]. These MSC-derived exosomes carry molecular signals that support neuronal survival and regeneration, positioning them as a promising option for future stroke therapies. Ongoing clinical studies are investigating the potential of exosome-based therapies for ischemic stroke, aiming to advance their use in clinical settings [139].

### Exosomes in traumatic brain and spinal cord injury

CNS trauma caused by accidents remains a leading cause of disability and death worldwide, often resulting in prolonged sensory and functional recovery. Current treatments for traumatic brain injury (TBI) and spinal cord injury (SCI) are limited, and effective therapeutic options are lacking. However, recent experimental research highlights the potential of exosome-based therapies in mitigating the effects of CNS trauma and enhancing recovery outcomes [189]. One promising approach involves the intranasal delivery of bone marrow BMSCs-derived exosomes, loaded with phosphatase and tensin homolog (PTEN) siRNA. This method has shown significant efficacy in targeting spinal cord lesions and reducing the extent of spinal cord injury [53]. By silencing PTEN, a molecule known to inhibit neuronal regeneration, these exosomes facilitate axonal repair and functional recovery [98]. Exosomes isolated from microglia carrying miR-124 have been successfully delivered to TBI sites in animal models. Administration of miR-124 fosters M2 polarization of microglial cells, directing them toward an anti-inflammatory and regenerative phenotype. This process enhances hippocampal neurogenesis and improves cognitive and neurological functions after TBI [182]. Further research indicates that NSCs-derived exosomes can promote angiogenesis, stimulate neurogenesis, and reduce apoptosis in experimental TBI models, offering another promising therapeutic approach [192].

## Engineering exosomes with neurotherapeutic application

Surface engineering techniques can further enhance exosome’s ability to deliver nucleic acids specifically to the brain [93]. Although native EVs or exosomes can cross the BBB, systemic administration of unmodified EVs from various cell types often results in their primary accumulation in the Liver and spleen, with less than 1% reaching the brain [112, 172]. To improve brain targeting, exosomes can be engineered to enhance their accumulation in the brain. We have summarized the strategies for engineering exosomes for brain targeting and delivery that undergo clinical evaluations in Table 2. In this section, we describe some of the important approaches.

**Table 2:** Summary of recent developments in artificial exosomes for brain targeting and delivery

Carrier	Method	Cargo	Purpose	References
T7-Lamp2b-exosomes	Genetic engineering	Antisense miR-21	Targeted delivery for glioma	Kim et al. [77]
CD9-ApoB-exosomes		Empty	Targeted delivery in middle cerebral artery occlusion	Choi et al. [28]
RVG-Lamp2b-exosomes		MSC-derived	Targeted delivery for Alzheimer’s disease	Cui et al. [34]
		BACE1 siRNA		Alvarez-Erviti et al. [9]
		NGF pro and mRNA	Targeted delivery for ischemic stroke	Yang et al. [179]
		HMGB1-siRNA		Kim et al. [78]
		miR-124		Yang et al.
		Opioid receptor siRNA	Targeted delivery for inhibiting morphine relapse	Liu et al. [100]
		circDYM	Targeted delivery for depression	Yu et al. [185]
		Aptamer F5R1 or F5R2	Targeted delivery for Parkinson’s disease	Ren et al. [129]
RVG-CP05-CD63-exosomes	Anchor peptide	EXOPMO	Targeted delivery to the brain	Gao et al. [46]
CXCR4-exosomes	Genetic engineering	TRAIL	Targeted delivery for ischemic stroke	Li et al. [90]
RGD-C1C2		NPC-derived		Tian et al. [160]
c(RGDyK) peptide	Click chemistry	Curcumin		Tian et al. [159]
NRP-1 peptide	Click chemistry	Curcumin	Targeted delivery for glioblastoma	Thirumalai et al. [162]
AN2	Genetic engineering	STAT3 siRNA		Liang et al. [94]
AN2; CD133 RNA aptamers	Amphiphilic molecule bridge	Temozolomide and *O*^6^-benzylguanine		Liang et al. [95]
Nefmut-scFv-exosomes	Genetic engineering	Anti-CD24	Targeted delivery for Parkinson’s disease	Stott et al. [149]
D47-SST exosomes	Genetic engineering	miR29b-2	Targeted delivery for Alzheimer’s disease	Lin et al. [97]

### T7-exosomes

Recently, engineering exosomes with specific ligands have emerged as a promising strategy for targeted gene therapy in GBM (Table 2). By incorporating a transferrin receptor (TfR)-binding peptide, T7, onto the exosome surface, researchers have enhanced targeting of TfR-rich GBM cells, such as C6 cells, compared to unmodified or RVG-modified exosomes [56]. Moreover, genetic modification of exosomal surface proteins, expressing lysosome-associated membrane glycoprotein 2b (Lamp2b), can enhance their binding affinity to brain-specific targets while preserving the structural integrity of the exosomes. When T7-Lamp2b-Exo, loaded with anti-miR-21, was administered intravenously, it effectively reduced miR-21 levels in GBM tumors, leading to significant tumor suppression with reduced side effects [77]. In a comparative study, Kim et al. evaluated exosomes decorated with the T7 peptide (T7-exo) against those modified with the RVG peptide for delivering antisense miRNA oligonucleotides targeting miR-21 (AMO-21) to GBM in the brain. The T7-exo demonstrated superior delivery efficiency in both in vitro and in vivo settings. However, despite this enhanced efficiency, both T7-exo/AMO-21 and RVG-exo/AMO-21 exhibited similar therapeutic effects on brain tumors following systemic injection, highlighting an intriguing aspect that requires further investigation [77]. The T7 peptide’s effectiveness is attributed to its strong binding affinity to TfR and the elevated expression of TfRs in brain tumors, making it a valuable tool for targeted tumor therapy [72] (Table 2).

### RVG-exosomes

RVG is a peptide sequence from the rabies virus glycoprotein, which naturally targets the nicotinic acetylcholine receptor and neural cell adhesion molecules on neuronal cells. Scientists have successfully Fused a 29-amino-acid peptide from the RVG with Lamp2b (RVG-Lamp2b-Exos), creating exosomes with a specific binding affinity for nicotinic acetylcholine receptors [9]. These RVG-Exos, loaded with siRNA targeting BACE1, successfully deliver their cargo to neurons, microglia, and oligodendrocytes in the brain following intravenous administration [9]. Similarly, RVG-modified MSCs-derived exosomes specifically targeted brain regions affected by amyloid-beta, significantly increasing their accumulation in the cortex and hippocampus threefold and alleviating neuroinflammation in a mouse model of AD [34]. When these RVG-modified MSC exosomes were loaded with miR-124, they effectively delivered the miRNA to injury sites . Emerging research is also investigating the use of exosomes with Lamp2b-RVG on their surface, where TD of these exosomes to the brain in a mouse model of PD attenuated neurotoxicity and neuroinflammation [133]. Additionally, the use of RVG-modified exosomes for delivering HMGB1 siRNA into ischemic brain tissue led to reduced HMGB1 levels and smaller infarct sizes [78]. Moreover, RVG-Exos carrying circDYM effectively suppressed microglial activation and astrocyte dysfunction, leading to improved depressive symptoms [185]. Additionally, the systemic delivery of recombinant human nerve growth factor (NGF) protein and its mRNA via RVG-Exos facilitated translation within the ischemic cortex, reducing ischemic injury. This delivery method improved brain function by modulating microglial polarization, supporting inflammatory cell survival, and increasing the population of doublecortin-positive cells [179]. Moreover, RVG-Exos encapsulating the aptamer F5R2, which targets fibrillar α-synuclein, successfully cleared α-synuclein aggregates from cultured neurons, prevented neuronal death and synaptic protein loss, and alleviated associated motor impairments [129]. A recent study demonstrated the successful use of engineered exosomes expressing the neuron-targeting RVG peptide on their surface to deliver siRNA against the opioid receptor mu (MOR) into the brain as a therapeutic strategy for morphine addiction [100]. The RVG-modified exosomes efficiently encapsulated MOR siRNA, which was shown to associate with AGO2 within the exosomal cargo. These exosomes enabled specific and effective delivery of MOR siRNA into Neuro2A cells and across the BBB in mice. Notably, treatment with the siRNA-loaded RVG exosomes significantly reduced MOR mRNA and protein expression in brain tissues, leading to a marked suppression of morphine relapse behavior [100]. Finally, a study introduced CP05, a peptide identified through phage display, which bound specifically to the exosomal surface protein CD63. The peptide enabled efficient targeting, loading, and capture of exosomes from various sources, including patient-derived ones, without altering the exosomal membrane. In therapeutic applications, exosomes loaded with CP05-modified phosphorodiamidate morpholino oligomers (EXOPMO) significantly increased dystrophin protein levels by 18-fold in the quadriceps of dystrophin-deficient *Mdx* mice compared to non-exosomal CP05-PMO. Further enhancement using a muscle-targeting peptide on EXOPMO led to improved dystrophin expression and functional recovery, with no observable toxicity [46]. These findings highlighted CP05 as a promising tool for engineering exosomes for targeted, non-toxic NAD and gene modulation in vivo. Altogether, these findings underscore the potential of RVG-Exos as a powerful tool for targeted brain therapies, addressing both NDDs and functional recovery post-injury (Table 2).

### Nefmut-scFv-exosomes

scFv (Single-Chain Variable Fragment) is a genetically engineered antibody fragment consisting of the variable regions of the heavy (VH) and light (VL) chains of an antibody, connected by a short peptide linker. This structure preserves the antigen-binding specificity of a full-length antibody while being significantly smaller, making scFvs ideal for therapeutic and diagnostic applications. Engineered EVs from transduced neuronal cells can be precisely targeted by co-expressing the Exo-anchoring protein (Nefmut)-scFv fusion protein, which includes membrane-binding proteins and ligands specific to the target cells. For example, fusing a humanized anti-CD24 scFv enables exosomes to target CD24-expressing cells, enhancing therapeutic effectiveness in conditions like PD [149].

### AN2-exosomes

The low-density lipoprotein receptor (LDLR)-related protein (LRP-1), expressed on brain capillary endothelial cells and glioma cells, is crucial for the transcytosis of endogenous proteins and small molecules into the brain. Leveraging this pathway, researchers have developed exosomes Functionalized with angiopoietin 2 (AN2), which has a high affinity for LRP-1, to enhance brain delivery. By displaying AN2 these engineered exosomes can exploit receptor-mediated transcytosis to efficiently cross the BBB. This TD system not only enhances the accumulation of therapeutic cargo such as siRNAs, chemotherapeutic agents, or neuroprotective molecules in brain tissues but also minimizes off-target effects. Liang and colleagues created AN2-functionalized exosomes (AN2-Exos) for TD of STAT3-siRNA, demonstrating improved brain targeting [94, 95] (Table 2). Moreover, dual-targeting exosomes, incorporating both AN2 and CD133 RNA aptamers, were effective in internalizing cargo in U87MG cells and GBM stem cells [94, 95]. These studies suggest that AN2-modified exosomes have substantial potential for improving brain therapies by enabling more efficient and TD of therapeutic agents to brain cells (Table 2).

### SST/CCK exosomes

Somatostatin (SST) and Cholecystokinin (CCK) are naturally occurring neuropeptides in the brain involved in neural signaling. By engineering exosomes to display SST or CCK, researchers aim to improve targeting specificity toward cells expressing SST or CCK receptors, such as neurons or glial cells involved in neurodegenerative or psychiatric diseases. Recently, these exosomes have been employed for targeted brain delivery of microRNA-29b-2 and further engineered to express CD47 proteins [97]. CD47, known for its role in inhibiting phagocytosis, is essential for protecting exosomes from immune clearance and ensuring their safe passage to the brain following BBB translocation. CD47 is a transmembrane protein commonly known as the “don’t eat me” signal because it binds to Signal Regulatory Protein alpha (SIRPα) receptors on immune cells such as macrophages and microglia. This interaction inhibits the phagocytic activity of these cells, allowing exosomes to avoid immune clearance and enhance their stability and targeting efficiency within the CNS [75]. By targeting somatostatin receptors, the delivery system effectively reduced presenilin 1 expression and β-amyloid accumulation in the brains of AD mouse models [97].

### Other strategies for enhancing exosome brain targeting

In addition to their natural properties, exosomes can be engineered for enhanced targeting through surface modification using tetraspanin superfamily proteins like CD63, CD9, and CD81. These proteins can be modified to present specific cell-targeting sequences by incorporating targeting components into their extracellular loops [16]. For example, the LDLR-mediated transcytosis pathway can be harnessed by engineering exosomes to express ApoB through conjugation with the tetraspanin CD9. In this study, exosomes were produced from Expi293F cells transiently transfected with either CD9 or a modified CD9 containing the ApoB targeting peptide (CD9/LEL170-ApoB). Results showed that CD9-ApoB exosomes accumulated prominently in cortical blood vessels, unlike the unmodified CD9 exosomes, which were not detected in the vasculature. Furthermore, the presence of CD9-ApoB exosomes in the brain was significantly higher and sustained up to 24 h, indicating enhanced targeting and prolonged retention within brain tissue [28].

The BBB has Limited strategies for treating brain metastases of breast cancer. MSCs-derived exosomes can bypass the BBB and deliver gene therapy to enhance chemotherapeutic efficacy. This study used exosomes modified with C-X-C chemokine receptor type 4 (CXCR4) and TNF-related apoptosis-inducing ligand (TRAIL) to target cancer cells. In a mouse model, exosome-CXCR4^+^TRAIL combined with carboplatin showed significant antitumor activity, suggesting a promising strategy for treating brain metastases. Further research is needed to assess the vector’s effectiveness on the BBB and its safety in animal models [90]. Another study investigates the potential of CXCR4-overexpressing bone marrow stem cells-derived exosomes in promoting vascular function and neural repair following ischemic stroke. Exosomes (ExoCXCR4) were isolated and characterized, and their effects were evaluated using a rat model of middle cerebral artery occlusion. Results showed that ExoCXCR4 significantly improved neurological function, promoted endothelial cell proliferation, and enhanced angiogenesis. The exosomes also played an antiapoptotic role via the Wnt-3a/β-catenin pathway. These findings suggest that ExoCXCR4 could serve as a potential neuroprotective therapy for ischemic stroke [90].

Moreover, strategies like the engineering of exosomes to incorporate BBB-crossing peptides like internalizing RGD (iRGD), have been shown to improve brain targeting [159] (Table 2). The iRGD is a tumor-penetrating peptide that binds to αvβ3/αvβ5 integrins and is cleaved to expose a CendR motif, which interacts with neuropilin-1 (NRP-1) to enhance tissue penetration, including BBB crossing [162]. Engineered exosomes modified with the cyclic peptide c(RGDyK) [cyclo(Arg-Gly-Asp-D-Tyr-Lys)] have demonstrated the ability to selectively target ischemic brain lesions by recognizing integrins overexpressed at injury sites. When loaded with curcumin, these Crgd-Exo effectively reduced neuroinflammation and cellular apoptosis [159]. Similarly, another targeting strategy using a fusion protein, RGD-C1C2 (comprising an Arg-Gly-Asp motif fused to the lactadherin-derived C1C2 domain), enabled exosomes to localize to ischemic regions in the brain and exert anti-inflammatory effects.

## Recent exosome-based clinical trials for neurological disease

Recent exosome-based clinical trials for neurological diseases have shown promising results. The clinical trial NCT03384433 evaluated the safety and efficacy of bone marrow MSCs (BM-MSCs)-derived exosomes in patients with acute ischemic stroke. The trial involved five male participants aged 40–80, who were given a single injection of 200 mg of BM-MSC-derived exosomes with miR-124, 1 month after their stroke. The primary goal was to assess safety, with a 12-month follow-up, and the secondary goal was to evaluate neurological improvements using the modified Rankin Scale. The results showed that the exosome therapy was safe with no adverse effects, and it led to significant neurological improvements in the participants, suggesting potential for treating acute ischemic stroke (Table 3).

**Table 3:** Clinical studies involving exosomes for neurological diseases

Exosome source	NIH identifier	Type	Purpose
Mesenchymal stem cell-derived exosomes	NCT03384433	Phase 2 enrollment: 5	Promoting recovery after acute ischemic stroke
Not specify	NCT04202770	Early-stage enrollment: 300	Treating depression, anxiety, and neurodegenerative dementia
Acupuncture patient-derived exosomes	NCT05326724	Phase 1 enrollment: 30	Treating post-stroke dementia
Not specified (LRRK2 positive exosomes)	NCT01860118	Phase 1 enrollment: 601	Treating Parkinson’s disease
Adipose mesenchymal stem cell-derived exosomes	NCT04388982	Phase 2 enrollment: 9	Treating Alzheimer’s disease
Not specify	NCT04202783	Early-stage	Treating depression, anxiety, and cognitive impairments
Not specified (antisense RG6042 loaded)	NCT03761849	Phase 3 enrollment: 791	Treating Huntington’s disease

NCT04202770 is a clinical trial investigating the safety and efficacy of exosome therapy combined with focused ultrasound in patients with refractory depression, anxiety, and neurodegenerative dementia. The trial uses MSCs-derived exosomes and focuses on improving cognitive function and reducing symptoms. Primary outcomes include changes in depression, anxiety, and cognitive function (Table 3).

NCT05326724 is a clinical trial titled “The Role of Acupuncture-Induced Exosome in Treating Post-Stroke Dementia,” conducted by China Medical University Hospital. The study aims to evaluate the therapeutic effects of acupuncture treatment-derived exosomes in individuals aged 50–70 with post-stroke dementia. Participants receive acupuncture to induce exosome release, and outcomes are assessed using standardized cognitive function scales. This trial is actively recruiting participants (Table 3).

The clinical trial NCT01860118, conducted by the University of Alabama at Birmingham, explored exosomal biomarkers associated with PD. The study aimed to identify proteins in exosomes that could serve as indicators of PD susceptibility and progression. Additionally, it investigated the effects of the LRRK2 kinase inhibitor sunitinib on LRRK2 expression and phosphorylation in exosomes from PD patients. While the study has concluded, detailed results have not been published (Table 3).

NCT04388982 is a Phase I/II clinical trial conducted at a single center. The study aimed to evaluate the safety and efficacy of allogenic human adipose MSCs-derived exosomes (ahaMSCs-Exos) in treating AD. Participants aged 50 and older received intranasal administrations of ahaMSCs-Exos twice weekly for 12 weeks. The trial utilized a three-arm, open-label design with escalating dosages to assess dose-limiting toxicity. The study concluded in April 2022, with findings indicating no adverse events and suggesting potential therapeutic benefits of ahaMSCs-Exos for AD (Table 3).

The clinical trial NCT04202783 investigates the combined use of focused ultrasound (FUS) and exosome therapy to treat various neurological conditions. The study aims to enhance the delivery of exosomes with therapeutic potential into specific brain regions using FuS, thereby improving treatment efficacy for depression, anxiety, and cognitive impairments associated with neurodegenerative diseases. The primary outcome measures include evaluating the safety of this combined approach and assessing potential adverse effects such as death, severe intraventricular hemorrhage, cystic periventricular leukomalacia, or other brain injuries, along with major neurodevelopmental impairments at 36 months of corrected age. Secondary outcomes involve short-term safety analyses and evaluations of therapeutic efficacy. This trial is being conducted by Neurological Associates of West Los Angeles and is currently recruiting participants (Table 3).

NCT03761849 was a Phase III study evaluating the efficacy and safety of intrathecally administered RO7234292 (tominersen) in patients with manifest Huntington’s disease. Conducted by Hoffmann-La Roche, the trial enrolled 791 participants aged 25–65, who were randomized to receive either tominersen or a placebo. The study was terminated in March 2021 after a pre-planned data review indicated that tominersen did not demonstrate a favorable benefit-to-risk profile (Table 3). These trials indicate a growing interest in harnessing exosomes for brain-targeted therapies.

## Challenges in the clinical translation of exosome-based therapeutics

Although exosome-based therapeutics hold great promise for targeted brain delivery, several key challenges must be addressed to support clinical translation. Regulatory agencies such as the FDA and EMA need to establish clear guidelines and quality standards to ensure the safe and effective development of these therapies. Successful translation also depends on the availability of accurate and standardized labeling and tracking methods to assess exosome biodistribution, pharmacokinetics, and stability. Current approaches, such as lipophilic dyes and donor cell genetic modifications, face limitations in precision and clinical relevance, highlighting the need for non-invasive and regulatory-compliant imaging technologies.

Ethical concerns regarding exosome sourcing are also critical. While MSC-derived exosomes are generally preferred for their safety and therapeutic potential, tumor-derived exosomes pose oncogenic risks. Another major issue is immunogenicity, while exosomes are generally considered biocompatible, modifications such as surface engineering or loading of foreign cargos can trigger immune responses, especially upon repeated administration. Moreover, the full composition and off-target effects of exosomal cargo must be carefully evaluated, particularly for long-term applications in humans. Another significant concern is their unpredictable biodistribution; despite surface targeting strategies, exosomes often accumulate in off-target organs like the liver, spleen, or lungs, limiting delivery efficiency to the brain. Additionally, scalable and standardized manufacturing remains a major hurdle. Conventional isolation methods like ultracentrifugation are inefficient at large scale and lack reproducibility, and the absence of widely accepted quality control and potency assays continues to impede regulatory approval and clinical use. These limitations underscore the need for stringent safety assessments, improved targeting strategies, and regulatory frameworks to guide their therapeutic use.

## Conclusion and prospective

Recent FDA approvals of RNA-based therapies for neurological and neuromuscular disorders highlight the growing potential of this modality. These include Patisiran (Onpattro) and Vutrisiran (Amvuttra), both siRNAs for treating hereditary transthyretin-mediated amyloidosis; Nusinersen (Spinraza), an ASO that modifies SMN2 splicing; and Risdiplam (Evrysdi), a small molecule that promotes exon 7 inclusion, both used in spinal muscular atrophy. Tofersen (Qalsody), an ASO, targets mutant SOD1 mRNA in ALS, and Milasen, a compassionate-use ASO, was developed for Batten disease (CLN7 variant). These cases demonstrate the versatility of RNA therapeutics in addressing genetic causes of neurological conditions. Based on our analysis, stem cells are the predominant source of exosomes used in neurological disease therapy, while exosomes derived from Glial cells remain comparatively underexplored. Genetic engineering is commonly employed to create artificial exosomes tailored for TD to the brain. Among various cargo types, RNA molecules are most frequently loaded into exosomes to achieve precise therapeutic targeting. These strategies have been investigated in the context of multiple neurological disorders, including Glioblastoma, Parkinson’s disease, and Ischemic Stroke, highlighting both the current progress and the potential for future innovation in this rapidly advancing field.

Exosomes offer multiple distinct advantages over both traditional nanocarriers and cell-based therapies, making them an increasingly attractive platform in nanomedicine. Compared to synthetic nanodelivery systems, exosomes are typically non-toxic and exhibit immunomodulatory properties, minimizing adverse immune reactions. Their natural ability to cross the BBB and target lesion sites addresses a major obstacle in treating neurological diseases. Moreover, exosomes inherently carry bioactive molecules that contribute to therapeutic effects beyond the delivered cargo. When compared to cell transplantation, exosomes present a safer and more controllable alternative. Lacking a nucleus, they cannot replicate in vivo, thereby eliminating the risk of teratoma formation. They also allow for rigorous quality control, as they can be sterilized by filtration and stored at low temperatures without loss of function. Exosomes penetrate the BBB more effectively through systemic injection than transplanted cells and possess a high surface-to-volume ratio, enabling enhanced modulation of ligand-mediated signaling. Their capacity for efficient drug loading and transcytosis-mediated tissue penetration further strengthens their therapeutic potential, positioning exosomes as a versatile and clinically viable drug delivery system. Furthermore, although the behavior of transplanted cells is often influenced by the surrounding microenvironment, exosomes act as more autonomous carriers of therapeutic cargo, which may lead to more predictable and consistent therapeutic outcomes.

A key limitation of using cell-derived exosomes for brain-TD is the limited understanding of their native cargo. While MSC-derived exosomes are commonly used (modified or not) the exact biomolecules they carry and their contribution to therapeutic effects remain unclear. This uncertainty can lead to off-target effects and low reproducibility. High-throughput RNA sequencing and proteomics are needed to better characterize exosome content and understand mechanisms of action. To address this, various methods like electroporation, sonication, and chemical treatments are used to remove endogenous cargo and load exosomes with specific therapeutic agents. It’s important to note that while these techniques can help create empty or “washed” exosomes, achieving a completely cargo-free exosome preparation can be challenging, and the methods used depend on the specific application and the properties of the cargo being removed or loaded.

As a prospective strategy to enhance BBB penetration, the conjugation of cell-penetrating peptides to exosomes holds significant promise. For example, Polyglutamine-binding peptide 1 has demonstrated the ability to enhance the delivery of therapeutic agents across the BBB, selectively reducing polyglutamine-expanded huntingtin protein aggregation. Notably, it does so without interfering with other amyloid proteins, such as β-amyloid, highlighting its high target specificity for neurodegenerative diseases [194]. Among the tested peptides, SynB3 exhibited superior delivery efficiency compared to the commonly used TAT peptide. Furthermore, integrating external stimuli, such as magnetic targeting [42] and ultrasound guidance [148], offers additional potential to improve the accumulation and penetration of exosomes into the brain, thereby advancing their application in precision neurotherapeutics.

## Data Availability

No datasets were generated or analysed during the current study. Data included in the article/referenced in the report.

## References

[1] Achar A, Myers R, Ghosh C. Drug delivery challenges in brain disorders across the blood–brain barrier: novel methods and future considerations for improved therapy. Biomedicines. 2021;9(12):1834. 34944650 10.3390/biomedicines9121834 PMC8698904

[2] Adachi T, Nakamura Y. Aptamers: a review of their chemical properties and modifications for therapeutic application. Molecules. 2019;24(23):4229. 31766318 10.3390/molecules24234229 PMC6930564

[3] Ahmad S, Srivastava RK, Singh P, Naik UP, Srivastava AK. Role of extracellular vesicles in glia-neuron intercellular communication. Front Mol Neurosci. 2022;15: 844194. 35493327 10.3389/fnmol.2022.844194 PMC9043804

[4] Ahmadzada T, Reid G, McKenzie DR. Fundamentals of siRNA and miRNA therapeutics and a review of targeted nanoparticle delivery systems in breast cancer. Biophys Rev. 2018;10(1):69–86. 29327101 10.1007/s12551-017-0392-1 PMC5803180

[5] Ahmed W, Kuniyan MS, Jawed AM, Chen L. Engineered extracellular vesicles for drug delivery in therapy of stroke. Pharmaceutics. 2023;15(9):2173. 37765144 10.3390/pharmaceutics15092173 PMC10537154

[6] Aljabali AAA, El-Tanani M, Tambuwala MM. Principles of CRISPR-Cas9 technology: advancements in genome editing and emerging trends in drug delivery. J Drug Deliv Sci Technol. 2024;92: 105338.

[7] Alcayaga-Miranda F, Varas-Godoy M, Khoury M. Harnessing the angiogenic potential of stem cell-derived exosomes for vascular regeneration. Stem Cells Int. 2016;2016:3409169. 27127516 10.1155/2016/3409169 PMC4834153

[8] Alqudah A, Aljabali AA, Gammoh OM, Tambuwala M. Advancements in neurotherapeutics: nanoparticles overcoming the blood–brain barrier for precise CNS targeting. J Nanopart Res. 2024;26:123.

[9] Alvarez-Erviti L, Seow Y, Yin H, Betts C, Lakhal S, Wood MJA. Delivery of siRNA to the mouse brain by systemic injection of targeted exosomes. Nat Biotechnol. 2011;29(4):341–5. 21423189 10.1038/nbt.1807

[10] Anesten F, Jansson J-O. Blood–brain shuttles—a new way to reach the brain? Nat Metab. 2021;3(8):1040–1. 34341567 10.1038/s42255-021-00428-1

[11] Apostolopoulos A, Kawamoto N, Chow SYA, Tsuiji H, Ikeuchi Y, Shichino Y, Iwasaki S. dCas13-mediated translational repression for accurate gene silencing in mammalian cells. Nat Commun. 2024;15:2205. 38467613 10.1038/s41467-024-46412-7 PMC10928199

[12] Armstrong MJ, Okun MS. Diagnosis and treatment of Parkinson’s disease: a review. JAMA. 2020;323(6):548–60. 32044947 10.1001/jama.2019.22360

[13] Argaw A.T, Asp L, Zhang J, Navrazhina K, Pham T, Mariani JN, Mahase S, Dutta DJ, Seto J, Kramer EG, Ferrara N, Sofroniew MV, John GR. Astrocyte-derived VEGF-A drives blood-brain barrier disruption in CNS inflammatory disease. J Clin Invest. 2012;122(7):2454–68. 10.1172/JCI60842 PMC3386814 22653056

[14] Argaw AT, Asp L (2018) REST, a master transcriptional regulator in neurodegenerative disease. Curr Opin Neurobiol 48:193–200 29351877 10.1016/j.conb.2017.12.008 PMC5892838

[15] Asadujjaman M, Jang D-J, Cho KH, Hwang SR, Jee J-P. Extracellular vesicles: the next frontier in regenerative medicine and drug delivery. Adv Exp Med Biol. 2020;1249:143–60. 32602096 10.1007/978-981-15-3258-0_10

[16] Bahadorani M, Nasiri M, Dellinger K, Aravamudhan S, Zadegan R. Engineering exosomes for therapeutic applications: decoding biogenesis, content modification, and cargo loading strategies. Int J Nanomedicine. 2024;19:7137–64. 39050874 10.2147/IJN.S464249 PMC11268655

[17] Bai L, Yu L, Ran M, Zhong X, Sun M, Xu M, Wang Y, Yan X, Lee RJ, Tang Y, Xie J. Harnessing the potential of exosomes in therapeutic interventions for brain disorders. Int J Mol Sci. 2025;26(6):2491. 40141135 10.3390/ijms26062491 PMC11942545

[18] Banks WA, Sharma P, Bullock KM, Hansen KM, Ludwig N, Whiteside TL. Transport of extracellular vesicles across the blood–brain barrier: brain pharmacokinetics and effects of inflammation. Int J Mol Sci. 2020;21(12):4407. 32575812 10.3390/ijms21124407 PMC7352415

[19] Bonafede R, Brandi J, Manfredi M, Scambi I, Schiaffino L, Merigo F, Turano E, Bonetti B, Marengo E, Cecconi D. The anti-apoptotic effect of ASC-exosomes in an in vitro ALS model and their proteomic analysis. Cells. 2019;8(9):1087. 31540100 10.3390/cells8091087 PMC6770878

[20] Bonafede R, Scambi I, Peroni D, Potrich V, Boschi F, Benati D, Bonetti B, Mariotti R. Exosome derived from murine adipose-derived stromal cells: neuroprotective effect on in vitro model of amyotrophic lateral sclerosis. Exp Cell Res. 2016;340(1):150–8. 26708289 10.1016/j.yexcr.2015.12.009

[21] Breitkreuz-Korff O, Tscheik C, Del Vecchio G, Dithmer S, Walther W, Orthmann A, Wolburg H, Haseloff RF, Schroeder L, Blasig IE. M01 as a novel drug enhancer for specifically targeting the blood–brain barrier. J Control Release. 2021;338:137–48. 34384796 10.1016/j.jconrel.2021.08.014

[22] Calabria E, Scambi I, Bonafede R, Schiaffino L, Peroni D, Potrich V, Capelli C, Schena F, Mariotti R. Ascs-exosomes recover coupling efficiency and mitochondrial membrane potential in an in vitro model of als. Front Neurosci. 2019;13:1070. 31680811 10.3389/fnins.2019.01070 PMC6811497

[23] Cano A, Fonseca E, Ettcheto M, Sánchez-López E, de Rojas I, Alonso-Lana S, Morato X, Souto EB, Toledo M, Boada M. Epilepsy in neurodegenerative diseases: related drugs and molecular pathways. Pharmaceuticals. 2021;14(10):1057. 34681281 10.3390/ph14101057 PMC8538968

[24] Chaves JCS, Dando SJ, White AR, Oikari LE. Blood–brain barrier transporters: an overview of function, dysfunction in Alzheimer’s disease and strategies for treatment. Biochim Biophys Acta Mol Basis Dis. 2024;1870(2): 166967. 38008230 10.1016/j.bbadis.2023.166967

[25] Chauhan I, Yasir M, Verma M, Singh AP. Nanostructured lipid carriers: a groundbreaking approach for transdermal drug delivery. Adv Pharm Bull. 2020;10(2):150. 32373485 10.34172/apb.2020.021 PMC7191226

[26] Chen H, Yao H, Chi J, Li C, Liu Y, Yang J, Yu J, Wang J, Ruan Y, Pi J, Xu JF. Engineered exosomes as drug and RNA co-delivery system: new hope for enhanced therapeutics? Front Bioeng Biotechnol. 2023;11:1254356. 37823027 10.3389/fbioe.2023.1254356 PMC10562639

[27] Choudhury R. Harnessing exosomes: the future of drug delivery across biological barriers. Asia Pac J Pharmacother Toxicol. 2025;5:1–10.

[28] Choi H, Choi K, Kim DH, Oh BK, Yim H, Jo S, Choi C. Strategies for targeted delivery of exosomes to the brain: advantages and challenges. Pharmaceutics. 2022;14(3):672. 35336049 10.3390/pharmaceutics14030672 PMC8948948

[29] Costa J, de Carvalho M. Emerging molecular biomarker targets for amyotrophic lateral sclerosis. Clin Chim Acta. 2016;455:7–14. 26774696 10.1016/j.cca.2016.01.011

[30] Cox B, Nicolaï J, Williamson B. The role of the efflux transporter, P-glycoprotein, at the blood–brain barrier in drug discovery. Biopharm Drug Dispos. 2023;44(1):113–26. 36198662 10.1002/bdd.2331

[31] Cramer SP, Simonsen HJ, Varatharaj A, Galea I, Frederiksen JL, Larsson HB. Permeability of the blood–brain barrier predicts no evidence of disease activity at 2 years after natalizumab or fingolimod treatment in relapsing-remitting multiple sclerosis. Ann Neurol. 2018;83(5):902–14. 29604233 10.1002/ana.25219 PMC6032831

[32] Crooke ST, Baker BF, Crooke RM, Liang X-H. Antisense technology: an overview and prospectus. Nat Rev Drug Discovery. 2021;20(6):427–53. 33762737 10.1038/s41573-021-00162-z

[33] Crooke ST, Wang S, Vickers TA, Shen W, Liang XH. Cellular uptake and trafficking of antisense oligonucleotides. Nat Biotechnol. 2017;35(3):230–7. 28244996 10.1038/nbt.3779

[34] Cui G-H, Guo H-D, Li H, Zhai Y, Gong Z-B, Wu J, Liu J-S, Dong Y-R, Hou S-X, Liu J-R. RVG-modified exosomes derived from mesenchymal stem cells rescue memory deficits by regulating inflammatory responses in a mouse model of Alzheimer’s disease. Immun Ageing. 2019;16(1):1–12. 31114624 10.1186/s12979-019-0150-2 PMC6515654

[35] Cukovic D, Bagla S, Ukasik D, Stemmer PM, Jena BP, Naik AR, Sood S, Asano E, Luat A, Chugani DC, Dombkowski AA. Exosomes in epilepsy of tuberous sclerosis complex: carriers of pro-inflammatory microRNAs. Noncoding RNA. 2021;7(3):40. 34287356 10.3390/ncrna7030040 PMC8293460

[36] D’Amico RS, Aghi MK, Vogelbaum MA, Bruce JN. Convection-enhanced drug delivery for glioblastoma: a review. J Neurooncol. 2021;151(3):415–27. 33611708 10.1007/s11060-020-03408-9 PMC8034832

[37] Damase TR, Sukhovershin R, Boada C, Taraballi F, Pettigrew RI, Cooke JP. The limitless future of RNA therapeutics. Front Bioeng Biotechnol. 2021;9: 628137. 33816449 10.3389/fbioe.2021.628137 PMC8012680

[38] Darras BT, Farrar MA, Mercuri E, Finkel RS, Foster R, Hughes SG, Bhan I, Farwell W, Gheuens S. An integrated safety analysis of infants and children with symptomatic spinal muscular atrophy (SMA) treated with nusinersen in seven clinical trials. CNS Drugs. 2019;33:919–32. 31420846 10.1007/s40263-019-00656-w PMC6776494

[39] Doherty C, Wilbanks B, Khatua S, Maher LJ. Aptamers in neuro-oncology: an emerging therapeutic modality. Neuro Oncol. 2024;26(1):38–54. 37619244 10.1093/neuonc/noad156 PMC10768989

[40] de Azambuja Borges CRL, Silva NO, Rodrigues MR, Marinho MAG, de Oliveira FS, Cassiana M, Horn AP, Parize AL, Flores DC, Clementin RM. Dimiristoylphosphatidylcholine/genistein molecular interactions: a physico-chemical approach to anti-glioma drug delivery systems. Chem Phys Lipid. 2019;225: 104828. 10.1016/j.chemphyslip.2019.104828 31550456

[41] Dhuri K, Bechtold C, Quijano E, Pham H, Gupta A, Vikram A, Bahal R. Antisense oligonucleotides: an emerging area in drug discovery and development. J Clin Med. 2020;9(6):2004. 32604776 10.3390/jcm9062004 PMC7355792

[42] Dwivedi P, Kiran S, Han S, Dwivedi M, Khatik R, Fan R, Mangrio FA, Du K, Zhu Z, Yang C. Magnetic targeting and ultrasound activation of liposome–microbubble conjugate for enhanced delivery of anticancer therapies. ACS Appl Mater Interfaces. 2020;12(21):23737–51. 32374147 10.1021/acsami.0c05308

[43] Fu S, Wang Y, Xia X, Zheng JC. Exosome engineering: current progress in cargo loading and targeted delivery. NanoImpact. 2020;20: 100261.

[44] Gadhave DG, Kokare CR. Nanostructured lipid carriers engineered for intranasal delivery of teriflunomide in multiple sclerosis: optimization and in vivo studies. Drug Dev Ind Pharm. 2019;45(5):839–51. 30702966 10.1080/03639045.2019.1576724

[45] Ganjeifar B, Morshed SF. Targeted drug delivery in brain tumors-nanochemistry applications and advances. Curr Top Med Chem. 2021;21(14):1202–23. 33185163 10.2174/1568026620666201113140258

[46] Gao X, Ran N, Dong X, Zuo B, Yang R, Zhou Q, Moulton HM, Seow Y, Yin H. Anchor peptide captures, targets, and loads exosomes of diverse origins for diagnostics and therapy. Sci Transl Med. 2018;10(444):eaat0195. 29875202 10.1126/scitranslmed.aat0195

[47] Gareev I, Beylerli O, Tamrazov R, Ilyasova T, Shumadalova A, Du W, Yang B. Methods of miRNA delivery and possibilities of their application in neuro-oncology. Noncoding RNA Res. 2023;8(4):661–74. 37860265 10.1016/j.ncrna.2023.10.002 PMC10582311

[48] Gawade A, Polshettiwar S, Hingalajia H, Prajapati BG, Singh A. Pharmacokinetics and pharmacodynamics of various novel formulations targeting Alzheimer’s disease. In: Alzheimer’s disease and advanced drug delivery strategies. Amsterdam: Elsevier; 2024. p. 391–402.

[49] Gholami L, Tafaghodi M, Abbasi B, Daroudi M, Kazemi Oskuee R. Preparation of superparamagnetic iron oxide/doxorubicin loaded chitosan nanoparticles as a promising glioblastoma theranostic tool. J Cell Physiol. 2019;234(2):1547–59. 30145790 10.1002/jcp.27019

[50] Ghorai SM, Deep A, Magoo D, Gupta C, Gupta N. Cell-penetrating and targeted peptides delivery systems as potential pharmaceutical carriers for enhanced delivery across the blood–brain barrier (BBB). Pharmaceutics. 2023;15(7):1999. 37514185 10.3390/pharmaceutics15071999 PMC10384895

[51] Goel K, Ploski JE. RISC-y business: limitations of short hairpin RNA-mediated gene silencing in the brain and a discussion of CRISPR/Cas-based alternatives. Front Mol Neurosci. 2022;15: 914430. 35959108 10.3389/fnmol.2022.914430 PMC9362770

[52] Gu J, Al-Bayati K, Ho EA. Development of antibody-modified chitosan nanoparticles for the targeted delivery of siRNA across the blood–brain barrier as a strategy for inhibiting HIV replication in astrocytes. Drug Deliv Transl Res. 2017;7:497–506. 28315051 10.1007/s13346-017-0368-5

[53] Guo S, Perets N, Betzer O, Ben-Shaul S, Sheinin A, Michaelevski I, Popovtzer R, Offen D, Levenberg S. Intranasal delivery of mesenchymal stem cell derived exosomes loaded with phosphatase and tensin homolog siRNA repairs complete spinal cord injury. ACS Nano. 2019;13(9):10015–28. 31454225 10.1021/acsnano.9b01892

[54] Gupta A, Mishra A, Puri N. Peptide nucleic acids: advanced tools for biomedical applications. J Biotechnol. 2017;259:148–59. 28764969 10.1016/j.jbiotec.2017.07.026 PMC7114329

[55] Gurung S, Perocheau D, Touramanidou L, Baruteau J. The exosome journey: from biogenesis to uptake and intracellular signalling. Cell Communication and Signaling. 2021;19(1):47. 33892745 10.1186/s12964-021-00730-1 PMC8063428

[56] Han L, Huang R, Liu S, Huang S, Jiang C. Peptide-conjugated PAMAM for targeted doxorubicin delivery to transferrin receptor overexpressed tumors. Mol Pharm. 2010;7(6):2156–65. 20857964 10.1021/mp100185f

[57] Hartl N, Gabold B, Uhl P, Kromer A, Xiao X, Fricker G, Mier W, Liu R, Merkel OM. ApoE—functionalization of nanoparticles for targeted brain delivery—a feasible method for polyplexes? Drug Deliv Transl Res. 2024;14(6):1660–77. 38087181 10.1007/s13346-023-01482-w PMC11052808

[58] Hu Y, Gaillard PJ, Rip J, Hammarlund-Udenaes M. Blood-to-brain drug delivery using nanocarriers. In: Drug delivery to the brain: physiological concepts, methodologies and approaches. Cham: Springer; 2022. p. 501–26.

[59] Hu Y, Hammarlund-Udenaes M, Fridén M. Understanding the influence of nanocarrier-mediated brain delivery on therapeutic performance through pharmacokinetic-pharmacodynamic modeling. J Pharm Sci. 2019;108(10):3425–33. 31163187 10.1016/j.xphs.2019.05.029

[60] Huo X, Zhang Y, Jin X, Li Y, Zhang L. A novel synthesis of selenium nanoparticles encapsulated PLGA nanospheres with curcumin molecules for the inhibition of amyloid β aggregation in Alzheimer’s disease. J Photochem Photobiol, B. 2019;190:98–102. 30504054 10.1016/j.jphotobiol.2018.11.008

[61] Huttunen J, Peltokangas S, Gynther M, Natunen T, Hiltunen M, Auriola S, Ruponen M, Vellonen KS, Huttunen KM. L-type amino acid transporter 1 (LAT1/Lat1)-utilizing prodrugs can improve the delivery of drugs into neurons, astrocytes and microglia. Sci Rep. 2019;9(1):12860. 31492955 10.1038/s41598-019-49009-z PMC6731241

[62] Iachetta G, Falanga A, Molino Y, Masse M, Jabès F, Mechioukhi Y, Laforgia V, Khrestchatisky M, Galdiero S, Valiante S. gH625-liposomes as tool for pituitary adenylate cyclase-activating polypeptide brain delivery. Sci Rep. 2019;9(1):9183. 31235716 10.1038/s41598-019-45137-8 PMC6591382

[63] Inglut CT, Sorrin AJ, Kuruppu T, Vig S, Cicalo J, Ahmad H, Huang H-C. Immunological and toxicological considerations for the design of liposomes. Nanomaterials. 2020;10(2):190. 31978968 10.3390/nano10020190 PMC7074910

[64] Jankovska N, Matej R. Molecular pathology of ALS: what we currently know and what important information is still missing. Diagnostics. 2021;11(8):1365. 34441299 10.3390/diagnostics11081365 PMC8391180

[65] Jankovičová J, Sečová P, Michalková K, Antalíková J. Tetraspanins, more than markers of extracellular vesicles in reproduction. Int J Mol Sci. 2020;21(20):7568. 33066349 10.3390/ijms21207568 PMC7589920

[66] Jeppesen DK, Zhang Q, Franklin JL, Coffey RJ. Extracellular vesicles and nanoparticles: emerging complexities. Trends Cell Biol. 2023;33(8):667–81. 36737375 10.1016/j.tcb.2023.01.002 PMC10363204

[67] Jiang Y, Liu J, Chen L, Jin Y, Zhang G, Lin Z, Du S, Fu Z, Chen T, Qin Y. Serum secreted miR-137-containing exosomes affects oxidative stress of neurons by regulating OXR1 in Parkinson’s disease. Brain Res. 2019;1722: 146331. 31301273 10.1016/j.brainres.2019.146331

[68] Johnsen KB, Burkhart A, Melander F, Kempen PJ, Vejlebo JB, Siupka P, Nielsen MS, Andresen TL, Moos T. Targeting transferrin receptors at the blood–brain barrier improves the uptake of immunoliposomes and subsequent cargo transport into the brain parenchyma. Sci Rep. 2017;7(1):1–13. 28871203 10.1038/s41598-017-11220-1 PMC5583399

[69] Johnsen KB, Burkhart A, Thomsen LB, Andresen TL, Moos T. Targeting the transferrin receptor for brain drug delivery. Prog Neurobiol. 2019;181: 101665. 31376426 10.1016/j.pneurobio.2019.101665

[70] Juan T, Fürthauer M. Biogenesis and function of ESCRT-dependent extracellular vesicles. Semin Cell Dev Biol. 2018;74:66–77. 28807885 10.1016/j.semcdb.2017.08.022

[71] Kalluri R, LeBleu VS. The biology, function, and biomedical applications of exosomes. Science. 2020;367(6478):eaau6977. 32029601 10.1126/science.aau6977 PMC7717626

[72] Kang Z, Zeng C, Tian L, Wang T, Yang S, Cheng Q, Zhang J, Meng Q, Zhang C, Meng Z. Transferrin receptor targeting segment T7 containing peptide gene delivery vectors for efficient transfection of brain tumor cells. Drug Deliv. 2022;29(1):2375–85. 35866298 10.1080/10717544.2022.2102696 PMC9310815

[73] Karami Z, Saghatchi Zanjani MR, Rezaee S, Rostamizadeh K, Hamidi M. Neuropharmacokinetic evaluation of lactoferrin-treated indinavir-loaded nanoemulsions: remarkable brain delivery enhancement. Drug Dev Ind Pharm. 2019;45(5):736–44. 30640551 10.1080/03639045.2019.1569039

[74] Katakowski M, Buller B, Zheng X, Lu Y, Rogers T, Osobamiro O, Shu W, Jiang F, Chopp M. Exosomes from marrow stromal cells expressing miR-146b inhibit glioma growth. Cancer Lett. 2013;335(1):201–4. 23419525 10.1016/j.canlet.2013.02.019 PMC3665755

[75] Khalaji A, Yancheshmeh FB, Farham F, Khorram A, Sheshbolouki S, Zokaei M, Vatankhah F, Soleymani-Goloujeh M. Don’t eat me/eat me signals as a novel strategy in cancer immunotherapy. Heliyon. 2023;9(10): e20507. 37822610 10.1016/j.heliyon.2023.e20507 PMC10562801

[76] Khongkow M, Yata T, Boonrungsiman S, Ruktanonchai UR, Graham D, Namdee K. Surface modification of gold nanoparticles with neuron-targeted exosome for enhanced blood–brain barrier penetration. Sci Rep. 2019;9(1):8278. 31164665 10.1038/s41598-019-44569-6 PMC6547645

[77] Kim G, Kim M, Lee Y, Byun JW, Lee M. Systemic delivery of microRNA-21 antisense oligonucleotides to the brain using T7-peptide decorated exosomes. J Control Release. 2020;317:273–81. 31730913 10.1016/j.jconrel.2019.11.009

[78] Kim M, Kim G, Hwang DW, Lee M. Delivery of high mobility group box-1 siRNA using brain-targeting exosomes for ischemic stroke therapy. J Biomed Nanotechnol. 2019;15(12):2401–12. 31748020 10.1166/jbn.2019.2866

[79] Klinkovskij A, Shepelev M, Isaakyan Y, Aniskin D, Ulasov I. Advances of genome editing with CRISPR/Cas9 in neurodegeneration: the right path towards therapy. Biomedicines. 2023;11(12):3333. 38137554 10.3390/biomedicines11123333 PMC10741756

[80] Kotowska AM, Fay M, Watts JA, Gilmore IS, Scurr DJ, Howe A, Capka V, Perez CE, Doud D, Patel S, Umbarger M, Langer R, Alexander MR. Study on molecular orientation and stratification in RNA-lipid nanoparticles by cryogenic orbitrap secondary ion mass spectrometry. Commun Chem. 2025;8:160. 40404835 10.1038/s42004-025-01526-x PMC12098871

[81] Kong AH-Y, Wu AJ, Ho OK-Y, Leung MM-K, Huang AS, Yu Y, Zhang G, Lyu A, Li M, Cheung K-H. Exploring the potential of aptamers in targeting neuroinflammation and neurodegenerative disorders: opportunities and challenges. Int J Mol Sci. 2023;24(14):1178. 37511539 10.3390/ijms241411780 PMC10380291

[82] Kumar P, Parmar RG, Brown CR, Willoughby JLS, Foster DJ, Babu IR, Schofield S, Jadhav V, Charisse K, Nair JK, Rajeev KG, Maier MA, Egli M, Manoharan M. 5′-Morpholino modification of the sense strand of an siRNA makes it a more effective passenger. Chem Commun. 2019;55:5139–42. 10.1039/c9cc00977a 30977478

[83] Jimenez-Sanchez M, Licitra F, Underwood BR, Rubinsztein DC. Huntington’s disease: mechanisms of pathogenesis and therapeutic strategies. Cold Spring Harb Perspect Med. 2017;7(7): a024240. 27940602 10.1101/cshperspect.a024240 PMC5495055

[84] Larcher LM, Pitout IL, Keegan NP, Veedu RN, Fletcher S. DNAzymes: expanding the potential of nucleic acid therapeutics. Nucleic Acid Ther. 2023;33(3):178–92. 37093127 10.1089/nat.2022.0066 PMC10278027

[85] Lee M, Ban J-J, Kim KY, Jeon GS, Im W, Sung J-J, Kim M. Adipose-derived stem cell exosomes alleviate pathology of amyotrophic lateral sclerosis in vitro. Biochem Biophys Res Commun. 2016;479(3):434–9. 27641665 10.1016/j.bbrc.2016.09.069

[86] Lee ST, Im W, Ban JJ, Lee M, Jung KH, Lee SK, Chu K, Kim M. Exosome-based delivery of miR-124 in a Huntington's disease model. J Mov Disord. 2017; 10(1):45-52. 10.14802/jmd.16054. 10.14802/jmd.16054 10.14802/jmd.16054 PMC5288667 28122430

[87] Li H, Cao Y, Ye J, Yang Z, Chen Q, Liu X, Zhang B, Qiao J, Tang Q, Yang H. Engineering brain-derived neurotrophic factor mRNA delivery for the treatment of Alzheimer’s disease. Chem Eng J. 2023;466: 143152.

[88] Li T, Yang Y, Qi H, Cui W, Zhang L, Fu X, He X, Liu M, Li PF, Yu T. CRISPR/Cas9 therapeutics: progress and prospects. Sig Transduct Target Ther. 2023;8:36. 10.1038/s41392-023-01309-7 PMC9841506 36646687

[89] Li S, Li X, Wang N, Zhang C, Sang Y, Sun Y, Xia X, Zheng M. Brain targeted biomimetic siRNA nanoparticles for drug resistance glioblastoma treatment. J Control Release. 2024;376:67–78. 39368706 10.1016/j.jconrel.2024.10.004

[90] Li X, Zhang Y, Wang Y, Zhao D, Sun C, Zhou S, Xu D, Zhao J. Exosomes derived from CXCR4-overexpressing BMSC promoted activation of microvascular endothelial cells in cerebral ischemia/reperfusion injury. Neural Plast. 2020;2020:8814239. 33381162 10.1155/2020/8814239 PMC7762674

[91] Li Y, Xing L, Wang L, Liu X, Wu L, Ni M, Zhou Z, Li L, Liu X, Huang Y. Milk-derived exosomes as a promising vehicle for oral delivery of hydrophilic biomacromolecule drugs. Asian J Pharm Sci. 2023;18(2): 100797. 37035132 10.1016/j.ajps.2023.100797 PMC10073618

[92] Liang P, Shi H, Zhu W, Gui Q, Xu Y, Meng J, Guo X, Gong Z, Chen H. Silver nanoparticles enhance the sensitivity of temozolomide on human glioma cells. Oncotarget. 2017;8(5):7533. 27893419 10.18632/oncotarget.13503 PMC5352340

[93] Liang Y, Iqbal Z, Lu J, Wang J, Zhang H, Chen X, Duan L, Xia J. Cell-derived nanovesicle-mediated drug delivery to the brain: principles and strategies for vesicle engineering. Mol Ther. 2023;31(5):1207–24. 36245129 10.1016/j.ymthe.2022.10.008 PMC10188644

[94] Liang SF, Zuo FF, Yin BC, Ye BC. Delivery of siRNA based on engineered exosomes for glioblastoma therapy by targeting STAT3. Biomater Sci. 2022;10(6):1582–90. 35179533 10.1039/d1bm01723c

[95] Liang S, Xu H, Ye BC. Membrane-decorated exosomes for combination drug delivery and improved glioma therapy. Langmuir. 2022;38(1):299–308. 34936368 10.1021/acs.langmuir.1c02500

[96] Lin Z, Gu Y, Zhou R, Wang M, Guo Y, Chen Y, Ma J, Xiao F, Wang X, Tian X. Serum exosomal proteins F9 and TSP-1 as potential diagnostic biomarkers for newly diagnosed epilepsy. Front Neurosci. 2020;14:737. 32848539 10.3389/fnins.2020.00737 PMC7417627

[97] Lin EY, Hsu SX, Wu BH, Deng YC, Wuli W, Li YS, Lee JH, Lin SZ, Harn HJ, Chiou TW. Engineered exosomes containing microRNA-29b-2 and targeting the somatostatin receptor reduce presenilin 1 expression and decrease the β-amyloid accumulation in the brains of mice with Alzheimer’s disease. Int J Nanomedicine. 2024;19:4977–94. 38828204 10.2147/IJN.S442876 PMC11144417

[98] Liu M, Li H, Xiong G, et al. Mesenchymal stem cell exosomes therapy for the treatment of traumatic brain injury: mechanism, progress, challenges and prospects. J Transl Med. 2025;23:427. 40217480 10.1186/s12967-025-06445-y PMC11987214

[99] Liu C, Su C. Design strategies and application progress of therapeutic exosomes. Theranostics. 2019;9(4):1015–28. 30867813 10.7150/thno.30853 PMC6401399

[100] Liu Y, Li D, Liu Z, Zhou Y, Chu D, Li X, Jiang X, Hou D, Chen X, Chen Y, Yang Z, Jin L, Jiang W, Tian C, Zhou G, Zen K, Zhang J, Zhang Y, Li J, Zhang CY. Targeted exosome-mediated delivery of opioid receptor Mu siRNA for the treatment of morphine relapse. Sci Rep. 2015;5:17543. 26633001 10.1038/srep17543 PMC4668387

[101] Liu Z, Cheng L, Cao W, Shen C, Qiu Y, Li C, Xiong Y, Yang SB, Chen Z, Yin X, Zhang X. Present and future use of exosomes containing proteins and RNAs in neurodegenerative diseases for synaptic function regulation: a comprehensive review. Int J Biol Macromol. 2024;280(Pt 3): 135826. 39322147 10.1016/j.ijbiomac.2024.135826

[102] Liu T, Liu H, Xue S, Xiao L, Xu J, Tong S, Wei X. MiR129-5p-loaded exosomes suppress seizure-associated neurodegeneration in status epilepticus model mice by inhibiting HMGB1/TLR4-mediated neuroinflammation. Mol Biol Rep. 2024;51:292. 38332381 10.1007/s11033-024-09215-z PMC10853309

[103] Liu A, Wang X. The pivotal role of chemical modifications in mRNA therapeutics. Front Cell Dev Biol. 2022;10: 901510. 35912117 10.3389/fcell.2022.901510 PMC9326091

[104] Lombardo SM, Schneider M, Türeli AE, Günday Türeli N. Key for crossing the BBB with nanoparticles: the rational design. Beilstein J Nanotechnol. 2022;4(11):866–83. 10.3762/bjnano.11.72 PMC7277618 32551212

[105] Lu B, Lim JM, Yu B, Song S, Neeli P, Sobhani N, Pavithra K, Bonam SR, Kurapati R, Zheng J. The next-generation DNA vaccine platforms and delivery systems: advances, challenges and prospects. Front Immunol. 2024;15:1332939. 38361919 10.3389/fimmu.2024.1332939 PMC10867258

[106] Lu Z-G, Shen J, Yang J, Wang J-W, Zhao R-C, Zhang T-L, Guo J, Zhang X. Nucleic acid drug vectors for diagnosis and treatment of brain diseases. Signal Transduct Target Ther. 2023;8(1):39. 36650130 10.1038/s41392-022-01298-z PMC9844208

[107] Luque-Michel E, Sebastian V, Larrea A, Marquina C, Blanco-Prieto MJ. Co-encapsulation of superparamagnetic nanoparticles and doxorubicin in PLGA nanocarriers: development, characterization and in vitro antitumor efficacy in glioma cells. Eur J Pharm Biopharm. 2019;145:65–75. 31628997 10.1016/j.ejpb.2019.10.004

[108] Malakondaiah S, Julius A, Ponnambalam D, Gunthoti SS, Ashok J, Krishana PS, Rebecca J. Gene silencing by RNA interference: a review. Genome Instab Dis. 2024;5(5):225–41.

[109] Meccariello R, Bellenchi GC, Pulcrano S, D’Addario SL, Tafuri D, Mercuri NB, Guatteo E. Neuronal dysfunction and gene modulation by non-coding RNA in Parkinson’s disease and synucleinopathies. Front Cell Neurosci. 2024;17:1328269. 38249528 10.3389/fncel.2023.1328269 PMC10796818

[110] Micci M-A, Krishnan B, Bishop E, Zhang W-R, Guptarak J, Grant A, Zolochevska O, Tumurbaatar B, Franklin W, Marino C. Hippocampal stem cells promotes synaptic resistance to the dysfunctional impact of amyloid beta oligomers via secreted exosomes. Mol Neurodegener. 2019;14:1–22. 31200742 10.1186/s13024-019-0322-8 PMC6570890

[111] Miller CM, Donner AJ, Blank EE, Egger AW, Kellar BM, Østergaard ME, Seth PP, Harris EN. Stabilin-1 and Stabilin-2 are specific receptors for the cellular internalization of phosphorothioate-modified antisense oligonucleotides (ASOs) in the liver. Nucleic Acids Res. 2016;44(6):2782–94. 26908652 10.1093/nar/gkw112 PMC4824115

[112] Mirzaaghasi A, Han Y, Ahn S-H, Choi C, Park J-H. Biodistribution and pharmacokinectics of liposomes and exosomes in a mouse model of sepsis. Pharmaceutics. 2021;13(3):427. 33809966 10.3390/pharmaceutics13030427 PMC8004782

[113] Mirzaei S, Mahabady MK, Zabolian A, Abbaspour A, Fallahzadeh P, Noori M, Hashemi F, Hushmandi K, Daneshi S, Kumar AP. Small interfering RNA (siRNA) to target genes and molecular pathways in glioblastoma therapy: current status with an emphasis on delivery systems. Life Sci. 2021;275: 119368. 33741417 10.1016/j.lfs.2021.119368

[114] Mohammadinejad R, Dadashzadeh A, Moghassemi S, Ashrafizadeh M, Dehshahri A, Pardakhty A, Sassan H, Sohrevardi S-M, Mandegary A. Shedding light on gene therapy: carbon dots for the minimally invasive image-guided delivery of plasmids and noncoding RNAs-A review. J Adv Res. 2019;18:81–93. 30828478 10.1016/j.jare.2019.01.004 PMC6383136

[115] Morad G, Carman CV, Hagedorn EJ, Perlin JR, Zon LI, Mustafaoglu N, Park T-E, Ingber DE, Daisy CC, Moses MA. Tumor-derived extracellular vesicles breach the intact blood–brain barrier via transcytosis. ACS Nano. 2019;13(12):13853–65. 31479239 10.1021/acsnano.9b04397 PMC7169949

[116] Montazersaheb S, Hejazi MS, Nozad Charoudeh H. Potential of peptide nucleic acids in future therapeutic applications. Adv Pharm Bull. 2018;8(4):551–63. 30607328 10.15171/apb.2018.064 PMC6311635

[117] Nidhi S, Anand U, Oleksak P, Tripathi P, Lal JA, Thomas G, Kuca K, Tripathi V. Novel CRISPR-Cas systems: an updated review of the current achievements, applications, and future research perspectives. Int J Mol Sci. 2021;22(7):3327. 33805113 10.3390/ijms22073327 PMC8036902

[118] Østergaard ME, Jackson M, Low A, Chappell AE, Lee RG, Peralta RQ, Yu J, Kinberger GA, Dan A, Carty R, Tanowitz M, Anderson P, Kim TW, Fradkin L, Mullick AE, Murray S, Rigo F, Prakash TP, Bennett CF, Swayze EE, Gaus HJ, Seth PP. Conjugation of hydrophobic moieties enhances potency of antisense oligonucleotides in the muscle of rodents and non-human primates. Nucleic Acids Res. 2019;47(12):6045–58. 31076766 10.1093/nar/gkz360 PMC6614849

[119] Ozpolat B, Sood AK, Lopez-Berestein G. Liposomal siRNA nanocarriers for cancer therapy. Adv Drug Deliv Rev. 2014;66:110–6. 24384374 10.1016/j.addr.2013.12.008 PMC4527165

[120] Palanisamy CP, Pei J, Alugoju P, Anthikapalli NVA, Jayaraman S, Veeraraghavan VP, Gopathy S, Roy JR, Janaki CS, Thalamati D. New strategies of neurodegenerative disease treatment with extracellular vesicles (EVs) derived from mesenchymal stem cells (MSCs). Theranostics. 2023;13(12):4138. 37554286 10.7150/thno.83066 PMC10405853

[121] Pausch P, Al-Shayeb B, Bisom-Rapp E, Tsuchida CA, Li Z, Cress BF, Knott GJ, Jacobsen SE, Banfield JF, Doudna JA. CRISPR-CasΦ from huge phages is a hypercompact genome editor. Science. 2020;369(6501):333–7. 32675376 10.1126/science.abb1400 PMC8207990

[122] Pawar B, Vasdev N, Gupta T, Mhatre M, More A, Anup N, Tekade RK. Current update on transcellular brain drug delivery. Pharmaceutics. 2022;14(12):2719. 36559214 10.3390/pharmaceutics14122719 PMC9786068

[123] Perets N, Betzer O, Shapira R, Brenstein S, Angel A, Sadan T, Ashery U, Popovtzer R, Offen D. Golden exosomes selectively target brain pathologies in neurodegenerative and neurodevelopmental disorders. Nano Lett. 2019;19(6):3422–31. 30761901 10.1021/acs.nanolett.8b04148

[124] Qu L, Yi Z, Zhu S, Wang C, Cao Z, Zhou Z, Yuan P, Yu Y, Tian F, Liu Z. Programmable RNA editing by recruiting endogenous ADAR using engineered RNAs. Nat Biotechnol. 2019;37(9):1059–69. 31308540 10.1038/s41587-019-0178-z

[125] Quijano E, Bahal R, Ricciardi A, Saltzman WM, Glazer PM. Therapeutic peptide nucleic acids: principles, limitations, and opportunities. Yale J Biol Med. 2017;90(4):583–98. 29259523 PMC5733847

[126] Raman S, Mahmood S, Hilles AR, Javed MN, Azmana M, Al-Japairai KA. Polymeric nanoparticles for brain drug delivery-a review. Curr Drug Metab. 2020;21(9):649–60. 32384025 10.2174/1389200221666200508074348

[127] Rädler J, Gupta D, Zickler A, Andaloussi SE. Exploiting the biogenesis of extracellular vesicles for bioengineering and therapeutic cargo loading. Mol Ther. 2023;31(5):1231–50. 36805147 10.1016/j.ymthe.2023.02.013 PMC10188647

[128] Rehman FU, Liu Y, Zheng M, Shi B. Exosomes based strategies for brain drug delivery. Biomaterials. 2023;293: 121949. 36525706 10.1016/j.biomaterials.2022.121949

[129] Ren X, Zhao Y, Xue F, Zheng Y, Huang H, Wang W, Chang Y, Yang H, Zhang J. Exosomal DNA aptamer targeting α-synuclein aggregates reduced neuropathological deficits in a mouse Parkinson’s disease model. Mol Ther Nucleic Acids. 2019;17:726–40. 31437653 10.1016/j.omtn.2019.07.008 PMC6709346

[130] Rezaie J, Feghhi M, Etemadi T. A review on exosomes application in clinical trials: perspective, questions, and challenges. Cell Commun Signal. 2022;20(1):145. 36123730 10.1186/s12964-022-00959-4 PMC9483361

[131] Ronaldson PT, Davis TP. Targeting transporters: promoting blood–brain barrier repair in response to oxidative stress injury. Brain Res. 2015;14(1623):39–52. 10.1016/j.brainres.2015.03.018 PMC4569519 25796436

[132] Ruan Z, Pathak D, Venkatesan Kalavai S, Yoshii-Kitahara A, Muraoka S, Bhatt N, Takamatsu-Yukawa K, Hu J, Wang Y, Hersh S. Alzheimer’s disease brain-derived extracellular vesicles spread tau pathology in interneurons. Brain. 2021;144(1):288–309. 33246331 10.1093/brain/awaa376 PMC7880668

[133] Saikia B, Dhanushkodi A. Engineered exosome therapeutics for neurodegenerative diseases. Life Sci. 2024;356: 123019. 39209250 10.1016/j.lfs.2024.123019

[134] Salameh TS, Mortell WG, Logsdon AF, Butterfield DA, Banks WA. Disruption of the hippocampal and hypothalamic blood–brain barrier in a diet-induced obese model of type II diabetes: prevention and treatment by the mitochondrial carbonic anhydrase inhibitor, topiramate. Fluids Barriers CNS. 2019;16:1–17. 30616618 10.1186/s12987-018-0121-6 PMC6323732

[135] Salimi L, Seyedaghamiri F, Karimipour M, Mobarak H, Mardi N, Taghavi M, Rahbarghazi R. Physiological and pathological consequences of exosomes at the blood–brain-barrier interface. Cell Commun Signal. 2023;21:118. 37208741 10.1186/s12964-023-01142-z PMC10199515

[136] Sanadgol N, Zahedani SS, Sharifzadeh M, Khalseh R, Barbari GR, Abdollahi M. Recent updates in imperative natural compounds for healthy brain and nerve function: a systematic review of implications for multiple sclerosis. Curr Drug Targets. 2017;18(13):1499–517. 27829351 10.2174/1389450118666161108124414

[137] Sang A, Zhuo S, Bochanis A, Manautou JE, Bahal R, Zhong X-B, Rasmussen TP. Mechanisms of action of the US food and drug administration-approved antisense oligonucleotide drugs. BioDrugs. 2024;38(4):511–26. 38914784 10.1007/s40259-024-00665-2 PMC11695194

[138] Sardar Sinha M, Ansell-Schultz A, Civitelli L, Hildesjö C, Larsson M, Lannfelt L, Ingelsson M, Hallbeck M. Alzheimer’s disease pathology propagation by exosomes containing toxic amyloid-beta oligomers. Acta Neuropathol. 2018;136:41–56. 29934873 10.1007/s00401-018-1868-1 PMC6015111

[139] Schuldt BR, Kalagara R, Chennareddy S, Odland IC, Downes MH, Reford E, Vicari JM, Ali M, Bhimani AD, Putrino D, Kellner CP. Exosome-based therapy for ischemic stroke: a bibliometric analysis of current trends and future directions. World Neurosurg. 2023;171:e195–205. 36455847 10.1016/j.wneu.2022.11.125

[140] Shang R, Lee S, Senavirathne G, Lai EC. microRNAs in action: biogenesis, function and regulation. Nat Rev Genet. 2023;24(12):816–33. 37380761 10.1038/s41576-023-00611-y PMC11087887

[141] Sharma P, Srivastava P, Seth A, Tripathi PN, Banerjee AG, Shrivastava SK. Comprehensive review of mechanisms of pathogenesis involved in Alzheimer’s disease and potential therapeutic strategies. Prog Neurobiol. 2019;174:53–89. 30599179 10.1016/j.pneurobio.2018.12.006

[142] Shi Y, Zhen X, Zhang Y, Li Y, Koo S, Saiding Q, Kong N, Liu G, Chen W, Tao W. Chemically modified platforms for better RNA therapeutics. Chem Rev. 2024;124(3):929–1033. 38284616 10.1021/acs.chemrev.3c00611

[143] Silveira MM, Moreira GMSG, Mendonça M. DNA vaccines against COVID-19: perspectives and challenges. Life Sci. 2021;267: 118919. 33352173 10.1016/j.lfs.2020.118919 PMC7749647

[144] Silva AM, Almeida MI, Teixeira JH, et al. Dendritic cell-derived extracellular vesicles mediate mesenchymal stem/stromal cell recruitment. Sci Rep. 2017;7:1667. 28490808 10.1038/s41598-017-01809-x PMC5431789

[145] Singh RN, Singh NN. Mechanism of splicing regulation of spinal muscular atrophy genes. Adv Neurobiol. 2018;20:31–61. 29916015 10.1007/978-3-319-89689-2_2 PMC6026014

[146] Singh G, Monga V. Peptide nucleic acids: recent developments in the synthesis and backbone modifications. Bioorg Chem. 2023;141: 106860. 37748328 10.1016/j.bioorg.2023.106860

[147] Soares Martins T, Trindade D, Vaz M, Campelo I, Almeida M, Trigo G, da Cruz e Silva OA, Henriques AG. Diagnostic and therapeutic potential of exosomes in Alzheimer’s disease. J Neurochem. 2021;156(2):162–81. 32618370 10.1111/jnc.15112

[148] Sridharan B, Lim HG. Exosomes and ultrasound: the future of theranostic applications. Mater Today Bio. 2023;19: 100556. 36756211 10.1016/j.mtbio.2023.100556 PMC9900624

[149] Stott SRW, Hayat S, Carnwath T, Garas S, Sleeman JP, Barker RA. CD24 expression does not affect dopamine neuronal survival in a mouse model of Parkinson’s disease. PLoS ONE. 2017;12: e0171748. 28182766 10.1371/journal.pone.0171748 PMC5300212

[150] Sun J, Yuan Q, Guo L, Xiao G, Zhang T, Liang B, Yao R, Zhu Y, Li Y, Hu L. Brain microvascular endothelial cell-derived exosomes protect neurons from ischemia–reperfusion injury in mice. Pharmaceuticals. 2022;15(10):1287. 36297399 10.3390/ph15101287 PMC9608440

[151] Sun Z, Gao C, Gao D, Sun R, Li W, Wang F, Wang Y, Cao H, Zhou G, Zhang J, Shang J. Reduction in pericyte coverage leads to blood–brain barrier dysfunction via endothelial transcytosis following chronic cerebral hypoperfusion. Fluids Barriers CNS. 2021;18(1):21. 33952281 10.1186/s12987-021-00255-2 PMC8101037

[152] Sussman C, Liberatore RA, Drozdz MM. Delivery of DNA-based therapeutics for treatment of chronic diseases. Pharmaceutics. 2024;16(4):535. 38675196 10.3390/pharmaceutics16040535 PMC11053842

[153] Sweeney MD, Zhao Z, Montagne A, Nelson AR, Zlokovic BV. Blood–brain barrier: from physiology to disease and back. Physiol Rev. 2019;99(1):21–78. 30280653 10.1152/physrev.00050.2017 PMC6335099

[154] Tamba B, Streinu V, Foltea G, Neagu A, Dodi G, Zlei M, Tijani A, Stefanescu C. Tailored surface silica nanoparticles for blood–brain barrier penetration: preparation and in vivo investigation. Arab J Chem. 2018;11(6):981–90.

[155] Tansey MG, Wallings RL, Houser MC, Herrick MK, Keating CE, Joers V. Inflammation and immune dysfunction in Parkinson disease. Nat Rev Immunol. 2022;22(11):657–73. 35246670 10.1038/s41577-022-00684-6 PMC8895080

[156] Tan F, Li X, Wang Z, Li J, Shahzad K, Zheng J. Clinical applications of stem cell-derived exosomes. Signal Transduct Target Ther. 2024;9:17. 38212307 10.1038/s41392-023-01704-0 PMC10784577

[157] Takakusa H, Iwazaki N, Nishikawa M, Yoshida T, Obika S, Inoue T. Drug metabolism and pharmacokinetics of antisense oligonucleotide therapeutics: typical profiles, evaluation approaches, and points to consider compared with small molecule drugs. Nucleic Acid Ther. 2023;33(2):83–94. 36735616 10.1089/nat.2022.0054 PMC10066781

[158] Tasset A, Bellamkonda A, Wang W, Pyatnitskiy I, Ward D, Peppas N, Wang H. Overcoming barriers in non-viral gene delivery for neurological applications. Nanoscale. 2022;14(10):3698–719. 35195645 10.1039/d1nr06939j PMC9036591

[159] Tian T, Zhang HX, He CP, Fan S, Zhu YL, Qi C, Huang NP, Xiao ZD, Lu ZH, Tannous BA, Gao J. Surface functionalized exosomes as targeted drug delivery vehicles for cerebral ischemia therapy. Biomaterials. 2018;150:137–49. 29040874 10.1016/j.biomaterials.2017.10.012

[160] Tian T, Cao L, He C, Ye Q, Liang R, You W, Zhang H, Wu J, Ye J, Tannous BA, Gao J. Targeted delivery of neural progenitor cell-derived extracellular vesicles for anti-inflammation after cerebral ischemia. Theranostics. 2021;11:6507–21. 33995671 10.7150/thno.56367 PMC8120222

[161] Tsakiri M, Zivko C, Demetzos C, Mahairaki V. Lipid-based nanoparticles and RNA as innovative neuro-therapeutics. Front Pharmacol. 2022;13: 900610. 36016560 10.3389/fphar.2022.900610 PMC9395673

[162] Thirumalai A, Girigoswami K, Pallavi P, Harini K, Gowtham P, Girigoswami A. Cancer therapy with iRGD as a tumor-penetrating peptide. Bull Cancer. 2023;110(12):1288–300. 37813754 10.1016/j.bulcan.2023.08.009

[163] Waleed YR, Majed AA, Sherif ES, Osama AM, Anjana KN, Md Abdur R, Sabna K. Recent advances and future prospects of engineered exosomes as advanced drug and gene delivery systems. J Drug Deliv Sci Technol. 2025;106:106696.

[164] Wang D, Wu L-P. Nanomaterials for delivery of nucleic acid to the central nervous system (CNS). Mater Sci Eng, C. 2017;70:1039–46. 10.1016/j.msec.2016.04.011 27772703

[165] Wang L, Xiong X, Zhang L, Shen J. Neurovascular unit: a critical role in ischemic stroke. CNS Neurosci Ther. 2021;27(1):7–16. 33389780 10.1111/cns.13561 PMC7804897

[166] Wang Z, Xu W, Liu L, Zhu TF. A synthetic molecular system capable of mirror-image genetic replication and transcription. Nat Chem. 2016;8:698–704. 27325097 10.1038/nchem.2517

[167] Wang Y, Lu X, Wu X, Li Y, Tang W, Yang C, Liu J, Ding B. Chemically modified DNA nanostructures for drug delivery. Innovation. 2022;3(2): 100217. 35243471 10.1016/j.xinn.2022.100217 PMC8881720

[168] Wang J, Chen D, Ho EA. Challenges in the development and establishment of exosome-based drug delivery systems. J Control Release. 2021;329:894–906. 33058934 10.1016/j.jconrel.2020.10.020

[169] Wareham LK, Liddelow SA, Temple S, Benowitz LI, Di Polo A, Wellington C, Goldberg JL, He Z, Duan X, Bu G, Davis AA, Shekhar K, Torre AL, Chan DC, Canto-Soler MV, Flanagan JG, Subramanian P, Rossi S, Brunner T, Bovenkamp DE, Calkins DJ. Solving neurodegeneration: common mechanisms and strategies for new treatments. Mol Neurodegener. 2022;17(1):23. 35313950 10.1186/s13024-022-00524-0 PMC8935795

[170] Webb RL, Kaiser EE, Scoville SL, Thompson TA, Fatima S, Pandya C, Sriram K, Swetenburg RL, Vaibhav K, Arbab AS. Human neural stem cell extracellular vesicles improve tissue and functional recovery in the murine thromboembolic stroke model. Transl Stroke Res. 2018;9:530–9. 29285679 10.1007/s12975-017-0599-2 PMC6132936

[171] Weng Y, Li C, Yang T, Hu B, Zhang M, Guo S, Xiao H, Liang X-J, Huang Y. The challenge and prospect of mRNA therapeutics landscape. Biotechnol Adv. 2020;40: 107534. 32088327 10.1016/j.biotechadv.2020.107534

[172] Wiklander OP, Nordin JZ, O’Loughlin A, Gustafsson Y, Corso G, Mäger I, Vader P, Lee Y, Sork H, Seow Y. Extracellular vesicle in vivo biodistribution is determined by cell source, route of administration and targeting. J Extracell Vesicles. 2015;4(1):26316. 25899407 10.3402/jev.v4.26316 PMC4405624

[173] Wu D, Chen Q, Chen X, Han F, Chen Z, Wang Y. The blood–brain barrier: structure, regulation, and drug delivery. Signal Transduct Target Ther. 2023;8(1):217. 37231000 10.1038/s41392-023-01481-w PMC10212980

[174] Wu Y, Zhang B, Kebebe D, Guo L, Guo H, Li N, Pi J, Qi D, Guo P, Liu Z. Preparation, optimization and cellular uptake study of tanshinone I nanoemulsion modified with lactoferrin for brain drug delivery. Pharm Dev Technol. 2019;24(8):982–91. 31107131 10.1080/10837450.2019.1621897

[175] Xiao L, Zhao Y, Yang M, Luan G, Du T, Deng S, Jia X. A promising nucleic acid therapy drug: DNAzymes and its delivery system. Front Mol Biosci. 2023;10:1270101. 37753371 10.3389/fmolb.2023.1270101 PMC10518456

[176] Xin H, Wang F, Li Y, Lu QE, Cheung WL, Zhang Y, Zhang ZG, Chopp M. Secondary release of exosomes from astrocytes contributes to the increase in neural plasticity and improvement of functional recovery after stroke in rats treated with exosomes harvested from microRNA 133b-overexpressing multipotent mesenchymal stromal cells. Cell Transplant. 2017;26(2):243–57. 27677799 10.3727/096368916X693031 PMC5303172

[177] Xu Z, Zeng S, Gong Z, Yan Y. Exosome-based immunotherapy: a promising approach for cancer treatment. Mol Cancer. 2020;19:1–16. 33183286 10.1186/s12943-020-01278-3 PMC7661275

[178] Yang J, Luo S, Zhang J, Yu T, Fu Z, Zheng Y, Xu X, Liu C, Fan M, Zhang Z. Exosome-mediated delivery of antisense oligonucleotides targeting α-synuclein ameliorates the pathology in a mouse model of Parkinson’s disease. Neurobiol Dis. 2021;148: 105218. 33296726 10.1016/j.nbd.2020.105218

[179] Yang J, Wu S, Hou L, Zhu D, Yin S, Yang G, Wang Y. Therapeutic effects of simultaneous delivery of nerve growth factor mRNA and protein via exosomes on cerebral ischemia. Mol Ther Nucleic Acids. 2020;21:512–22. 32682291 10.1016/j.omtn.2020.06.013 PMC7365960

[180] Yang L, Li S, Hou C, Wang Z, He W, Zhang W. Recent advances in mRNA-based therapeutics for neurodegenerative diseases and brain tumors. Nanoscale. 2025;17(7):3537–48. 39750745 10.1039/d4nr04394d

[181] Yang Y, Okada S, Sakurai M. Adenosine-to-inosine RNA editing in neurological development and disease. RNA Biol. 2021;18(7):999–1013. 33393416 10.1080/15476286.2020.1867797 PMC8216190

[182] Yang Y, Ye Y, Kong C, Su X, Zhang X, Bai W, He X. MiR-124 enriched exosomes promoted the M2 polarization of microglia and enhanced hippocampus neurogenesis after traumatic brain injury by inhibiting TLR4 pathway. Neurochem Res. 2019;44:811–28. 30628018 10.1007/s11064-018-02714-z

[183] Yang H, Patel DJ. Structures, mechanisms and applications of RNA-centric CRISPR-Cas13. Nat Chem Biol. 2024;20:673–88. 38702571 10.1038/s41589-024-01593-6 PMC11375968

[184] You Y, Muraoka S, Jedrychowski MP, Hu J, McQuade AK, Young-Pearse T, Aslebagh R, Shaffer SA, Gygi SP, Blurton-Jones M, Poon WW, Ikezu T. Human neural cell type-specific extracellular vesicle proteome defines disease-related molecules associated with activated astrocytes in Alzheimer’s disease brain. J Extracell Vesicles. 2022;11(1): e12183. 35029059 10.1002/jev2.12183 PMC8758831

[185] Yu X, Bai Y, Han B, Ju M, Tang T, Shen L, Li M, Yang L, Zhang Z, Hu G. Extracellular vesicle-mediated delivery of circDYM alleviates CUS-induced depressive-like behaviours. J Extracell Vesicles. 2022;11(1): e12185. 35029057 10.1002/jev2.12185 PMC8758833

[186] Yu G, Wang X, Zhang Y, An Q, Wen Y, Li X, Yin H, Deng Z, Zhang H. Structure and function of a bacterial type III-E CRISPR-Cas7-11 complex. Nat Microbiol. 2022;7(12):2078–88. 36302881 10.1038/s41564-022-01256-z

[187] Yu T, Wang Z, Chen Y, Xiang Y, Wu M, Zhang M, Yin X, Chen Z. Blood–brain barrier (BBB) dysfunction in CNS diseases: paying attention to pericytes. CNS Neurosci Ther. 2022;31(5): e70422. 10.1111/cns.70422 PMC12079091 40371544

[188] Zha S, Liu H, Li H, Li H, Wong K-L, All AH. Functionalized nanomaterials capable of crossing the blood–brain barrier. ACS Nano. 2024;18(3):1820–45. 38193927 10.1021/acsnano.3c10674 PMC10811692

[189] Zhang Y, Chopp M, Meng Y, Katakowski M, Xin H, Mahmood A, Xiong Y. Effect of exosomes derived from multipluripotent mesenchymal stromal cells on functional recovery and neurovascular plasticity in rats after traumatic brain injury. J Neurosurg. 2015;122(4):856–67. 25594326 10.3171/2014.11.JNS14770 PMC4382456

[190] Zhang Y, Liu Y, Liu H, Tang WH. Exosomes: biogenesis, biologic function and clinical potential. Cell Biosci. 2019;9(1):1–18. 30815248 10.1186/s13578-019-0282-2 PMC6377728

[191] Zhang N, Bewick B, Schultz J, Tiwari A, Krencik R, Zhang A, Adachi K, Xia G, Yun K, Sarkar P, Ashizawa T. DNAzyme cleavage of CAG repeat RNA in polyglutamine diseases. Neurotherapeutics. 2021;18(3):1710–28. 34160773 10.1007/s13311-021-01075-w PMC8609077

[192] Zhong L, Wang J, Wang P, Liu X, Liu P, Cheng X, Cao L, Wu H, Chen J, Zhou L. Neural stem cell-derived exosomes and regeneration: cell-free therapeutic strategies for traumatic brain injury. Stem Cell Res Ther. 2023;14(1):198. 37553595 10.1186/s13287-023-03409-1 PMC10408078

[193] Zou Y, Sun X, Wang Y, Yan C, Liu Y, Li J, Zhang D, Zheng M, Chung RS, Shi B. Single siRNA nanocapsules for effective siRNA brain delivery and glioblastoma treatment. Adv Mater. 2020;32(24):2000416. 10.1002/adma.202000416 32374446

[194] Zuo L, Li W, Shi J, Su Y, Shuai H, Yu X. SynB3 conjugated QBP1 passes blood–brain barrier models and inhibits polyQ protein aggregation. Protein Pept Lett. 2022;29(1):110–20. 34939535 10.2174/0929866529666211221163930

